# Advancing medical imaging with language models: featuring a spotlight on ChatGPT

**DOI:** 10.1088/1361-6560/ad387d

**Published:** 2024-05-03

**Authors:** Mingzhe Hu, Joshua Qian, Shaoyan Pan, Yuheng Li, Richard L J Qiu, Xiaofeng Yang

**Affiliations:** 1 Department of Computer Science and Informatics, Emory University, Atlanta, GA, United States of America; 2 Department of Radiation Oncology, Winship Cancer Institute, School of Medicine, Emory University, Atlanta, GA, United States of America; 3 Department of Biomedical Engineering, Emory University, Atlanta, GA, United States of America

**Keywords:** medical imaging, ChatGPT, large language model, BERT, multimodal learning

## Abstract

This review paper aims to serve as a comprehensive guide and instructional resource for researchers seeking to effectively implement language models in medical imaging research. First, we presented the fundamental principles and evolution of language models, dedicating particular attention to large language models. We then reviewed the current literature on how language models are being used to improve medical imaging, emphasizing a range of applications such as image captioning, report generation, report classification, findings extraction, visual question response systems, interpretable diagnosis and so on. Notably, the capabilities of ChatGPT were spotlighted for researchers to explore its further applications. Furthermore, we covered the advantageous impacts of accurate and efficient language models in medical imaging analysis, such as the enhancement of clinical workflow efficiency, reduction of diagnostic errors, and assistance of clinicians in providing timely and accurate diagnoses. Overall, our goal is to have better integration of language models with medical imaging, thereby inspiring new ideas and innovations. It is our aspiration that this review can serve as a useful resource for researchers in this field, stimulating continued investigative and innovative pursuits of the application of language models in medical imaging.

## 1. Introduction

Medical physics has always been the forefront in implementing novel scientific discoveries and innovative technologies into the field of medicine. The origin of medical physics extends back to as early as 3000–2500 BC (Keevil 2012). In the history of modern medical physics, one of the most important milestones was the discovery of x-ray by Wilhelm Conrad Röntgen. The use of x-ray has revolutionized medical diagnostics and treatment. There are numerous other examples in the medical physics history book that significantly transformed medical practice, including the application of radiation therapy for cancer, the development of the gamma camera, the advent of positron emission tomography (PET) imaging, the invention of computed tomography (CT), and the advancement of magnetic resonance imaging (MRI), among others. Recently, there is a growing consensus that artificial intelligence (AI) is starting the fourth industrial revolution. Therefore, the integration of AI into medicine is of great interest to the medical physics community.

Over the past decade, deep learning (DL), a branch of AI, has experienced a significant surge in development and has found widespread applications, including autonomous vehicles, facial recognition, natural language processing, and medical diagnostics. DL models, categorized by data modality—vision, language, and sensor—have seen rapid development in vision and language owing to their adaptability and efficiency. Vision models, interpreting and processing visual data, have transformed medical imaging by improving diagnostic accuracy and efficiency in organ delineation (Pan *et al* 2022), advancing early disease detection (Lee *et al* 2022, Li *et al* 2022), achieving high-quality medical image synthesis (Lei *et al* 2018, Lei *et al* 2019, Yamashita and Markov 2020, Pan *et al* 2021) and facilitating personalized treatment plans (Florkow *et al* 2020). By utilizing the capabilities of vision models, the paradigm of medical imaging has shifted from conventional methods to AI-driven techniques, leading to improved patient outcomes and healthcare efficiency.

Conversely, language models (LMs), designed to understand, interpret, and generate human languages, have undergone significant advancements in recent years. These models have transformed various sectors by enabling more sophisticated text analysis, improving language translation systems, and enhancing user interaction with technology through natural language processing (NLP). Despite the extensive deployment and rapid progression of LMs in applications such as customer service chatbots, automated content creation, and language translation services, their integration into medical domains, particularly medical imaging, has not captured the researchers’ attention until recently due to several factors. Firstly, medical reports are replete with specialized terminology that requires precise interpretation and thorough analysis to ensure accurate and effective communication. Historically, LMs have encountered challenges in comprehending and utilizing the intricate medical terminology and subtleties essential for accurate medical diagnosis and effective patient care. Secondly, developing robust models requires large amounts of data and comprehensive corpora, which are difficult to obtain in the medical imaging domain due to stringent privacy regulations and the inherently confidential nature of medical records. This scarcity of accessible, annotated medical datasets has hindered the development and fine-tuning of LMs for medical applications. Thirdly, certain applications in medical imaging require an integration of vision and language models, known as vision-language models, which presents significant challenges, including ensuring coherent and meaningful correlations between image content and textual descriptions and adapting models to interpret medical images, which often contain highly specialized and nuanced information. Lastly, there is a noticeable deficit of comprehensive review literature that could help inspiring collaboration between researchers and medical professionals, which can lead to board applications for LMs in medical imaging.

**Figure 1.**
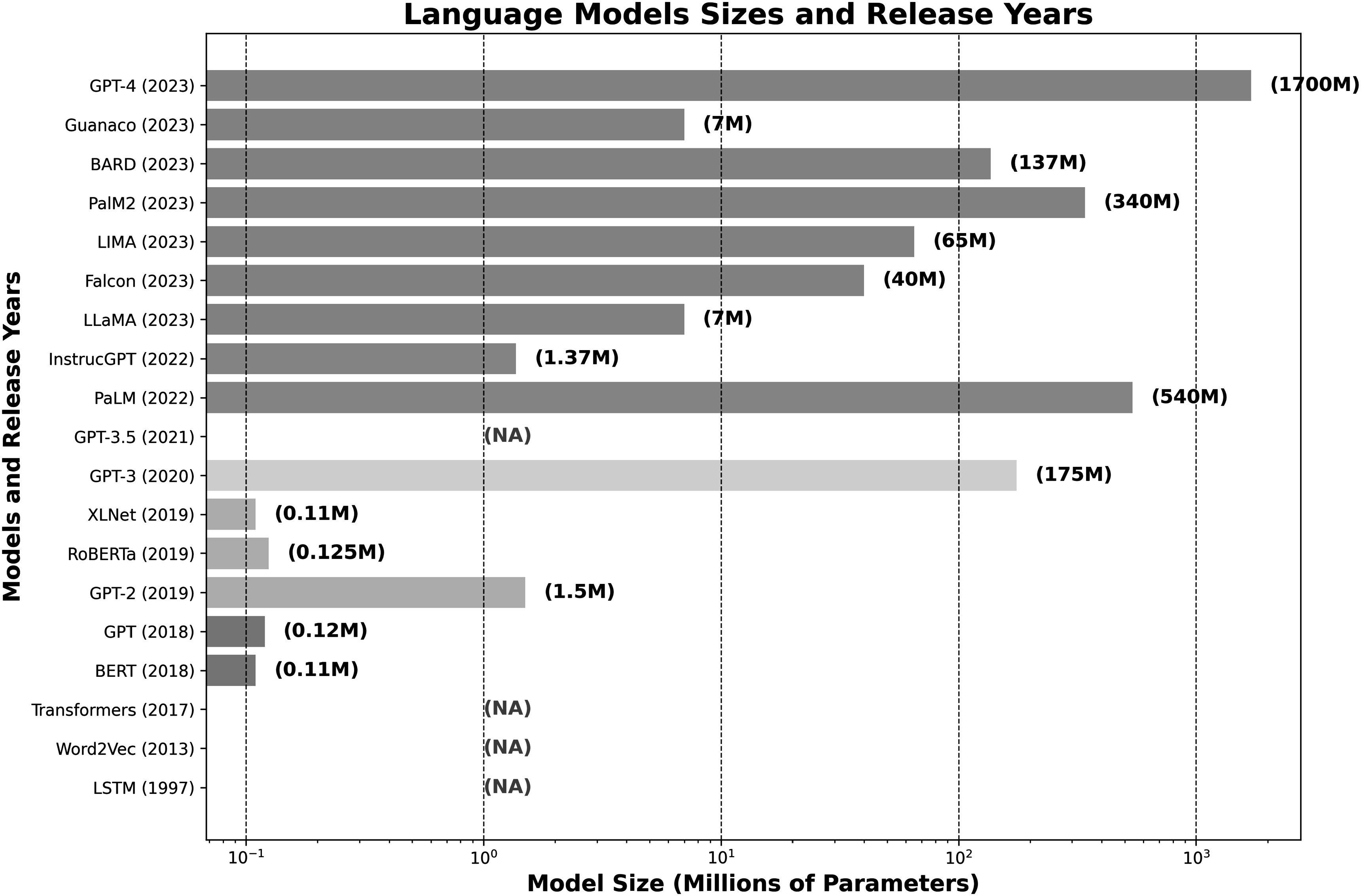
The bar chart illustrates the evolution of several influential language models, commencing with long short-term memory (LSTM) in 1997. Models released within the same year are represented by a unified color, while ‘NA’ denotes models whose sizes are either undisclosed or not applicable. The graph depicts a remarkable increase in the number of parameters of language models in recent times. While some models such as generative pre-trained transformer (GPT) by OpenAI and LLaMA (Large Language Model) by Meta AI have considerably scaled up in size to enhance their capabilities, others such as pathways language model (PaLM) by Google have employed diverse strategies to balance performance gains with efficiency, resulting in more compact model sizes.

The recent surge in interest towards LMs in the medical imaging is attributed to several key developments. One of the pivotal factors catalyzing this trend is the release of large-scale language models by several tech companies, as shown in figure 1, which exhibit exceptional proficiency in parsing and generating complex language structures. This breakthrough has prompted researchers to explore potential applications of LMs in medical imaging. Another contributing factor is the wide accessibility of these advanced pre-trained large models, engineered for immediate application or minimal customization, aligns well with the specialized requirements of medical imaging tasks. Such flexibility is advantageous for medical imaging applications, where specialized tasks can often be addressed with slight modifications to existing models by finetuning on domain-specific datasets, thus yielding more targeted and effective applications within medical contexts. Moreover, the emergence of multimodal large language models, GPT-4-V, has significantly reduced the complexity of adopting vision-language models in medical imaging. These models offer an integrated framework that merges visual and linguistic analytics capabilities, thus streamlining the interpretative process of medical images which frequently requires a combination of visual and textual analysis.

This review paper is presented in response to the challenges identified and aims to propel the use of language models in medical imaging. It is designed to lay the groundwork for foundational understanding and to motivate researchers in the medical imaging domain to pursue innovative uses of language models to advance the utility of medical imaging. Our review begins with a historical and conceptual survey of LMs, giving special emphasis to the significant strides in large language models and their expanding influence in this sphere. Subsequently, we dive into current research and applications specifically related to medical physics. We explore potential uses of LMs in medical imaging, including extracting findings from radiology reports (section 3.1), generating captions for images and videos (section 3.2), interpreting radiology diagnosis (section 3.3), classifying reports (section 3.4), creating detailed reports (section 3.5), learning from multimodal data (section 3.6), and answering visual questions (section 3.7).

**Figure 2.**
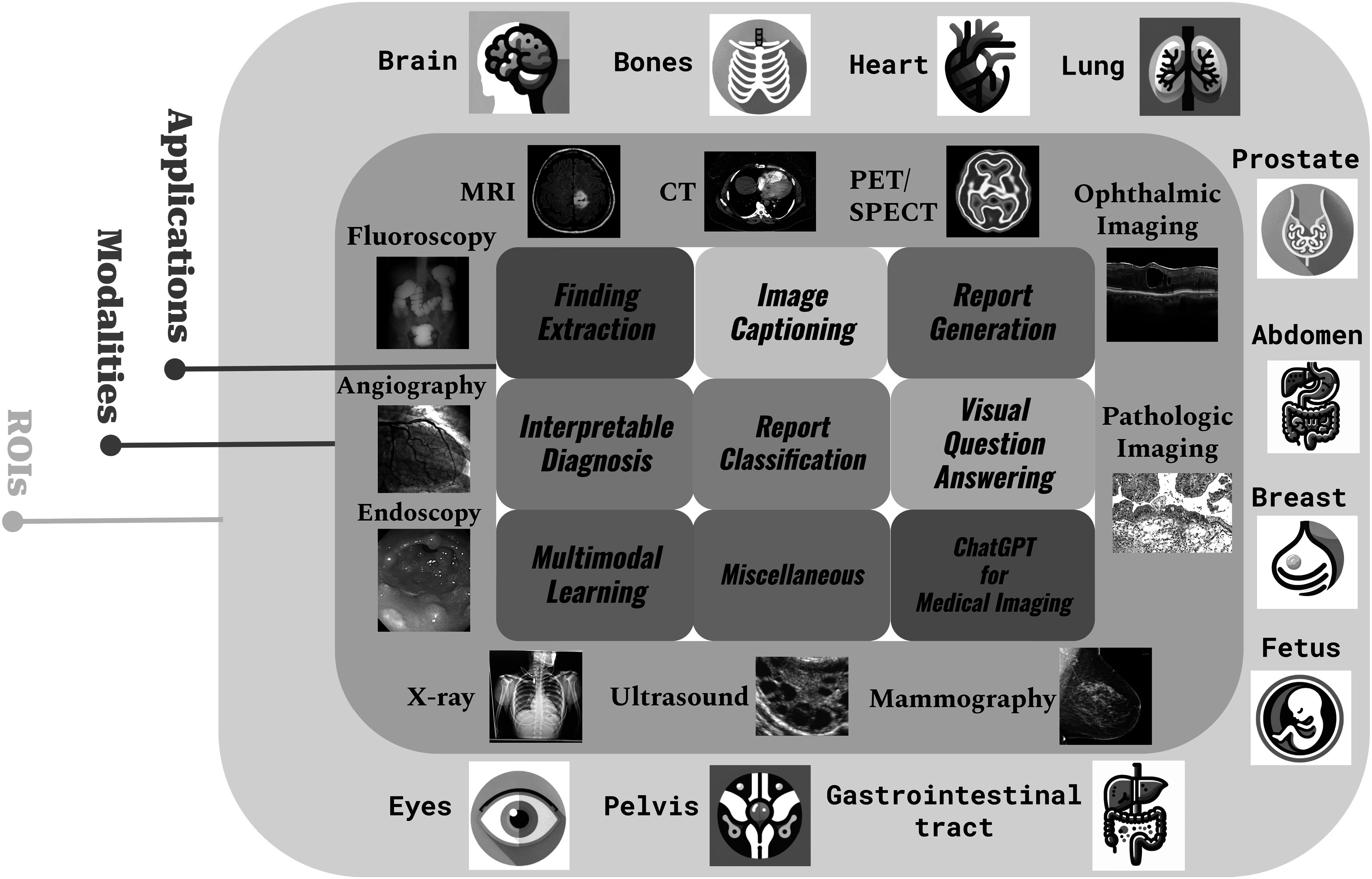
Comprehensive overview of topics in our review paper. The innermost part represents the specific applications discussed in our review, the middle circle encompasses the imaging modalities covered, and the outermost circle indicates the regions of interest (ROIs) addressed in the papers we reviewed.

In recognition of the profound advancements in natural language understanding and generation, we feature a dedicated section on ChatGPT (section 3.8). This section highlights ChatGPT’s powerful capabilities, the multi-modality of its derivative GPT-4-V, and the significant attention it has attracted from the academic community, making it one of the hottest topics in the field. The paper continues with an analysis of current literature, elucidating the diverse ways in which language models are enhancing medical imaging, as outlined in figure 2. We conclude with a thorough review of the current research landscape and a forward-looking discussion on potential future directions.

## 2. Basics of language models

### 2.1. Glossaries

Table 1 provides a list of common glossaries used in computer vison (CV) and NLP. This resource is intended for readers who may not have a solid background in machine learning or NLP, making it easier for them to understand our work. This table does not aim to be exhaustive.

**Table 1.** Common glossaries in natural language processing (NLP).

*Term*	Definition	*Term*	Definition	*Term*	Definition
*Machine learning (ML)*	A branch of computer science dealing with the simulation of intelligent behavior in computers	*Deep learning (DL)*	An advanced subset of ML that uses layered neural networks to analyze various factors of data	*Convolutional neural networks (CNNs)*	A type of deep neural network, primarily used to analyze visual imagery
*Natural language processing (NLP)*	A branch of AI focused on enabling computers to understand, interpret, and manipulate human language	*Transfer learning*	A method in ML where a model developed for a task is reused as the starting point for a model on a second task	*Reinforcement learning*	An area of ML concerned with how agents ought to take actions in an environment to maximize cumulative reward
*Vision-language models*	Models that understand and generate content combining visual and textual data	*Bag of words (BoW)*	A simple text representation model in NLP. It treats text as a collection of words regardless of their order or grammar	*Corpus*	A large collection of text documents or spoken language data used for training and testing NLP models
*Embedding*	Vector representations of words in a continuous space, capturing semantic relationships for NLP model improvement	*Feature engineering*	Selecting and transforming linguistic features from raw text for NLP model input preparation	*Hidden markov model (HMM)*	A statistical model in NLP representing sequence data, useful in tasks like speech recognition
*Information retrieval*	The process in NLP of retrieving relevant information from large text collections based on user queries	*Jaccard similarity*	A measure for comparing the similarity of two sets of words or documents based on shared elements	*Keyword extraction*	Automatically identifying and extracting key words or phrases from a document for summarization
*Lemmatization*	Reducing words to their base or root form in various word forms like singular/plural or verb tenses	*Machine translation*	An NLP task for automatically translating text from one language to another computationally	*Named entity recognition (NER)*	Identifying and classifying named entities (people, places, organizations) in text
*Ontology*	Formally representing knowledge by defining concepts and entities and their relationships in NLP	*Question answering*	An NLP task of generating accurate answers to questions posed in natural language	*Recurrence*	Using recurrent neural networks (RNNs) in NLP for processing data sequences in language modeling
*Sentiment analysis*	Determining the emotional tone or sentiment of text, typically as positive, negative, or neutral	*Tokenization*	Breaking text data into individual units (words, n-grams) for analysis in NLP tasks	*Unsupervised learning*	Training NLP models on data without explicit labels, allowing independent pattern learning
*Vector space model (VSM)*	A mathematical model transforming text into numerical vectors for similarity calculations in NLP	*Word sense disambiguation (WSD)*	Identifying the correct meaning of a word in context, especially for words with multiple meanings	*Zero-shot learning*	Training models to perform tasks they have not explicitly been trained on, used in various NLP applications

Some introductory-level articles in CV (Voulodimos *et al* 2018, Khan *et al* 2021) and NLP (Harrison and Sidey-Gibbons 2021, Lauriola *et al* 2022) may prepare readers with better backgrounds for our more in-depth reviewed topics.

### 2.2. Evaluation metrics

Comprehending the evaluation metrics for language models in medical imaging is of utmost importance. As our readership comprises a diverse group of professionals, ranging from machine learning researchers to medical physicists and physicians, an in-depth understanding of these metrics is critical for this broad audience. In this subsection, we present a list of these metrics and expound on their interpretation, laying the foundation for quantitatively understanding the performance of language models for medical imaging.

• *Accuracy*: This metric represents the overall percentage of correct predictions. In mathematical terms, it is expressed as:
  \begin{eqnarray*}\mathrm{Accuracy}\,=\,\frac{\text{Number of Correct Predictions}}{\text{Total Number of Predictions}}\end{eqnarray*}
• *Precision*: Precision is the ratio of correctly predicted positive observations to the total predicted positives. It is crucial for models where false positives are a significant concern.
  \begin{eqnarray*}\mathrm{Precision}\,=\,\frac{\text{True Positives}}{\text{True Positives}+\text{False Positives}}\end{eqnarray*}
• *Recall*: Recall measures the proportion of actual positives that are correctly identified. It is important in scenarios where missing a positive is more critical than falsely identifying a negative.
  \begin{eqnarray*}\mathrm{Recall}\,=\,\frac{\text{True Positives}}{\text{True Positives}+\text{False Negatives}}\end{eqnarray*}
• *F1 Score*: The F1 Score is the harmonic mean of precision and recall, giving a balance between these two metrics. It is particularly useful when the class distribution is uneven.
  \begin{eqnarray*}\mathrm{F}1\mathrm{Score}=2\times \frac{\mathrm{Precision}\times \mathrm{Recall}}{\mathrm{Precision}+\mathrm{Recall}}\end{eqnarray*}
• Area under the ROC curve (AUC): This metric measures a model’s ability to distinguish between classes. An AUC of 1 represents a perfect model, while 0.5 suggests no discriminative power.
• *BLEU (Bilingual evaluation understudy)*: Measures precision of n-gram overlap between generated and reference translations.
• *ROUGE (Recall-oriented understudy for gisting evaluation)*: Evaluates how well generated text captures the meaning of reference text. There are multiple ROUGE metrics (e.g. ROUGE-N, ROUGE-L), each with its own focus and calculation method.
• *METEOR*: A metric that incorporates exact word matching, synonym matching, and stemming to evaluate translation quality. Unlike BLEU, it accounts for semantic meaning and syntactic structure.
• *Perplexity*: Measures how well a language model predicts a sample. Lower perplexity indicates better model performance. Here, $p\left(x\right)$ is the probability of the sequence *x* according to the model.
  \begin{eqnarray*}\mathrm{Perplexity}\,=\,{2}^{-\sum p\left(x\right){\mathrm{log}}_{2}p\left(x\right)}\end{eqnarray*}
• *Word error rate (WER)*: Indicates the percentage of incorrect words in generated text compared to reference. Calculated as the number of substitutions, insertions, and deletions divided by total words in reference.
• *Mean reciprocal rank (MRR):* Averages the reciprocal ranks of the first relevant answer in a list of possibilities.
• *CIDEr (consensus-based image description evaluation):* CIDEr serves as an evaluative metric for image captioning that quantifies the quality and diversity of the generated captions. It gauges the term frequency-inverse document frequency (TF-IDF) scores for n-grams in both reference and generated captions and subsequently derives a consensus score. An elevated CIDEr score signifies an improvement in caption quality and diversity.
• *CLIPScore (contrastive language–image pre-training score):* CLIPScore is a tool designed to evaluate the correspondence between image descriptions and image content. It is particularly suitable for models such as CLIP. The tool leverages the CLIP model to embed the generated descriptions and images into a common semantic space and computes the cosine similarity between these embeddings. A higher CLIPScore indicates that the quality of the generated descriptions is better in terms of matching the visual content of the images.

### 2.3. The evolution of language models

#### 2.3.1. Pre-transformer era

Before the Transformer era, statistical and early neural network approaches dominated the landscape of language models. The most common of these were N-gram models, which used preceding words to predict the likelihood of the next word. However, these models were limited in their ability to capture long-range dependencies and were affected by data sparsity issues.

Later, probabilistic frameworks like hidden markov models (HMMs) (Rabiner 1989) were introduced, offering improved sequence modeling, but still struggled with the complexities of natural language. The introduction of recurrent neural networks (RNNs), particularly long short-term memory (LSTM) networks (Hochreiter and Schmidhuber 1997), marked a significant milestone as they could capture information over longer sequences, which earlier models could not. However, RNNs faced challenges like the vanishing gradient problem, which limited their efficacy in processing very long sequences.

This period before the Transformer models is crucial as it sets the foundation for developing them. The efforts of this era helped overcome several limitations in language modeling. They paved the way for developing more sophisticated models used today in various fields, such as medical imaging.

#### 2.3.2. Transformers

**Figure 3.**
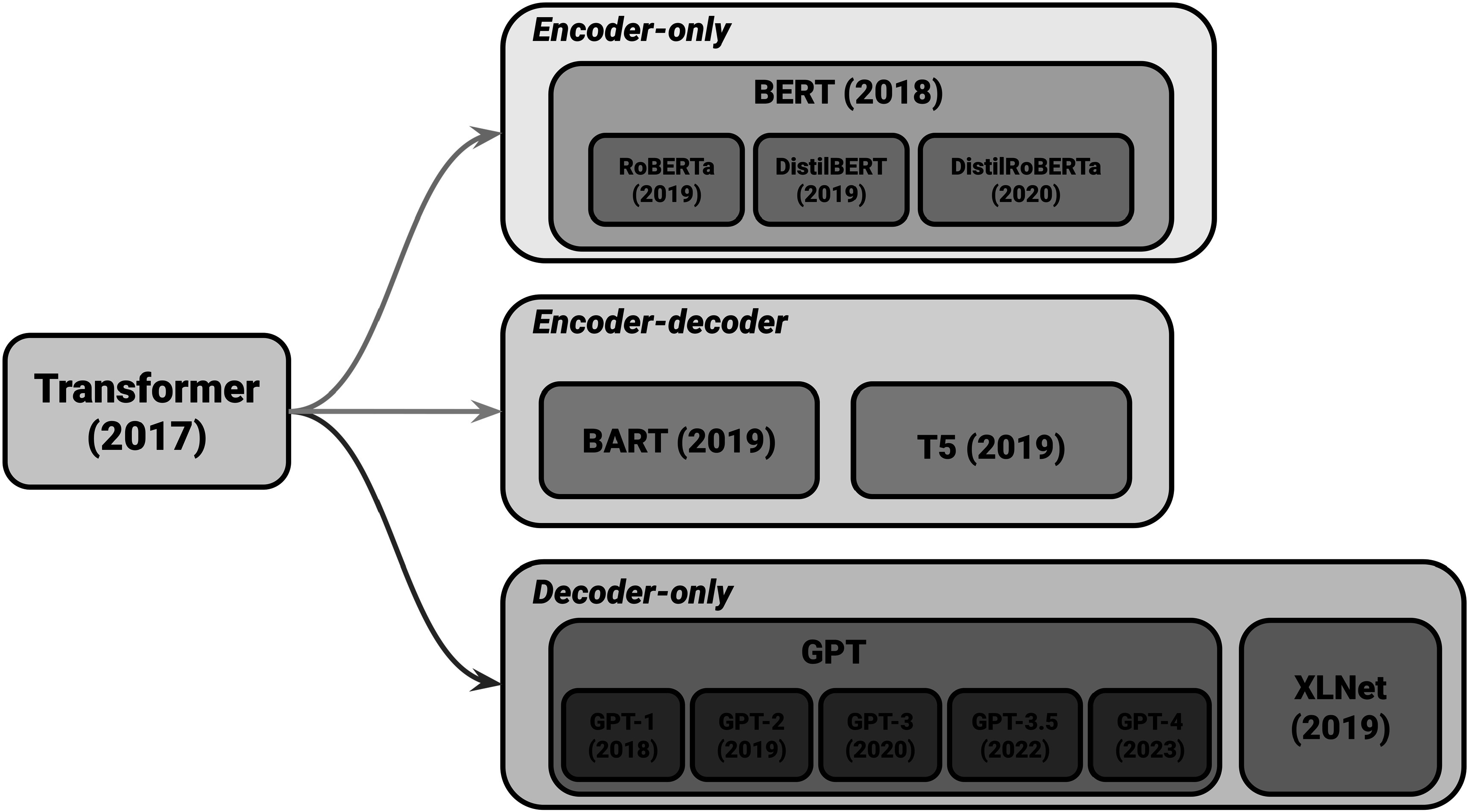
Transformer-based models. The graph illustrates models of different architectures—encoder-only (autoencoding), decoder-only (autoregressive), and encoder–decoder models.

Transformers, introduced by Vaswani *et al* (2017) have revolutionized the field of natural language processing with their innovative model architecture. They have surpassed traditional recurrent or convolutional encoder–decoder models. The key enhancement brought by attention mechanisms has significantly optimized the performance of encoder–decoder models. The Transformer model, the pioneering model that relies entirely on self-attention mechanisms for input and output representations, has outperformed both recurrent and convolutional neural networks. A notable advantage of the Transformer model is its ability to be trained in parallel, resulting in reduced training time. The encoder is composed of six identical blocks, each consisting of two sub-layers, a multi-head self-attention mechanism, and a fully connected feed-forward network. Similarly, the decoder comprises six identical blocks, each incorporating three sub-layers, including the addition of a multi-head self-attention sub-layer. The self-attention mechanism employed by the Transformer model utilizes a scaled dot-product attention approach. It involves multiplying the query and key vectors and applying a SoftMax function to obtain weightings for the output. Although both additive and dot-product attention are common attention functions, dot-product attention is favored for its superior space efficiency and faster computation, thanks to the utilization of highly optimized matrix multiplication code. A key aspect of the transformative impact of Transformers is their role as the foundational architecture for subsequent advanced language models. Models such as bidirectional encoder representations from transformers (BERT), generative pretrained transformer (GPT), T5 (Text-to-text transfer transformer) (Raffel *et al* 2020), bidirectional and auto-regressive transformers (BART) (Lewis *et al* 2019), and XLNet (Yang *et al* 2019) owe their genesis to the Transformer architecture shown as figure 3.

#### 2.3.3. BERT

BBERT (Lee and Toutanova 2018), a pre-trained LLM, has garnered significant attention in the NLP community for its outstanding performance across a range of tasks. Unlike its predecessors, which relied solely on unidirectional training, BERT adopts a bidirectional training approach. It predicts masked words within a sentence by considering both the left and right contexts. This unique approach enables the model to capture intricate relationships between words and phrases.

BERT’s pre-training process involves training on a diverse and extensive corpus of text, allowing it to learn the nuances and intricacies of natural language. As a result, BERT has achieved state-of-the-art performance on various benchmarks, including sentiment analysis, question-answering, and natural language inference. The impact of BERT on the field of NLP has been revolutionary, paving the way for the development of more advanced language models.

#### 2.3.4. PaLM

Parameterized language model (PaLM) (Peng *et al* 2019), an LLM proposed in 2019. Unlike traditional language models that use a fixed number of parameters to generate text, PaLM employs a dynamic parameterization approach. The model’s parameterization is adaptable, enabling it to generate text that is highly accurate and contextually relevant by considering the given context and input. The training of PaLM involves large-scale text data utilizing unsupervised learning techniques, facilitating its understanding of the underlying patterns and structures within natural language. Its versatility is evident in its ability to be fine-tuned for diverse NLP tasks, including text classification, question answering, and language modeling. PaLM’s remarkable feature lies in its proficiency in handling words that do not appear in the training data. The model can effectively generate contextually relevant words to replace out-of-vocabulary (OOV) words, improving its overall text generation performance.

#### 2.3.5. ChatGPT

ChatGPT, developed by OpenAI and based on the GPT (Radford *et al* 2018), has undergone a remarkable evolution through various iterations. Since its debut in June 2020, ChatGPT has progressively advanced with each version, showcasing OpenAI’s commitment to enhancing language model capabilities.

The trajectory of GPT’s development can be observed in its successive versions:

(1) *GPT-2*: This iteration marked a significant step forward from the original GPT, featuring a larger model size and an expanded training dataset. GPT-2 was notable for its improved text generation capabilities, setting new standards in language model performance.
(2) *GPT-*3: Representing a quantum leap in the series, GPT-3 was distinguished by its massive scale in terms of parameters and training data volume. It gained acclaim for its ability to generate text that remarkably mimics human writing, offering profound improvements in context understanding and nuanced response generation.
(3) *GPT-3.5*: As an intermediary update, GPT-3.5 bridged the gap between GPT-3 and GPT-4. It refined the capabilities of its predecessor, offering enhanced performance in text generation and understanding. This version continued to push the envelope in language processing, preparing the ground for the next significant advancement.
(4) *GPT-4*: The most recent in the series, GPT-4, has not only sustained the legacy of generating human-like text but has also introduced groundbreaking multimodal capabilities. This version can process and respond to both textual and visual stimuli, substantially broadening its applicability. GPT-4's (GPT-4-V’s) multimodality is a transformative feature, enabling more complex and dynamic interactions that closely resemble human cognitive abilities.

#### 2.3.6. LLaMA

LLaMA, an acronym for large language model meta AI, comprises a collection of foundational language models (Touvron *et al* 2023). The models underwent comprehensive training using unsupervised learning techniques, such as masked language modeling and next-sentence prediction, on an extensive corpus of tokens. The training data was sourced from publicly available datasets such as Wikipedia, Common Crawl, and OpenWebText. By training exclusively on publicly available data, The LLaMA team has demonstrated the feasibility of attaining cutting-edge performance without relying on proprietary datasets.

## 3. Language models for medical imaging

### 3.1. Finding extraction from radiology report

Extracting meaningful information from radiology reports is essential for secondary applications in clinical decision-making, research, and outcomes prediction. However, there remain several challenges in the field, such as reducing the workload of radiologists and improving communication with referring physicians. Furthermore, the task of extracting detailed semantic representations of radiological findings from reports requires a significant amount of laborious work and meticulous effort.

**Figure 4.**
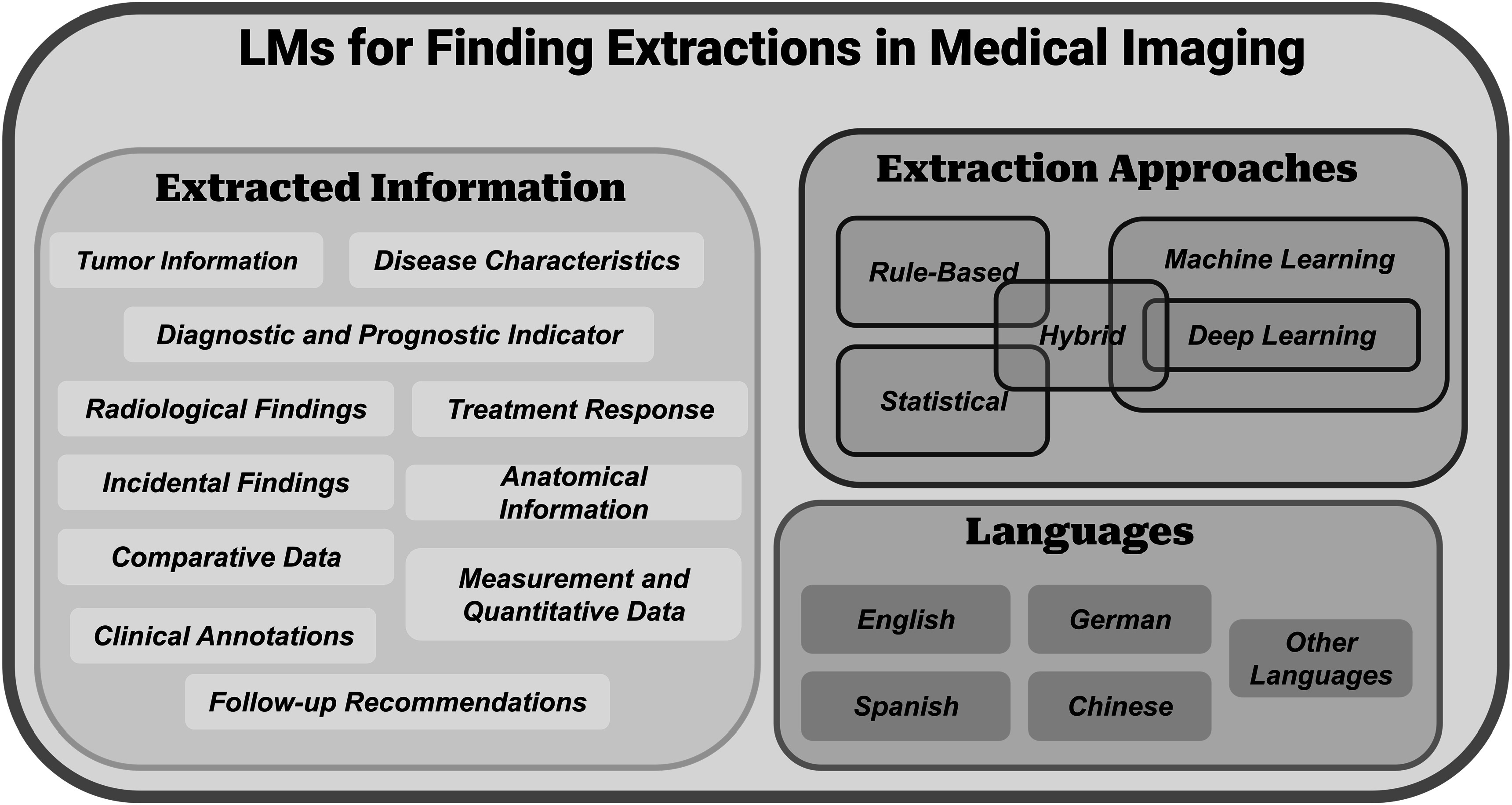
The schematic view of LMS for information extraction in medical imaging. We can categorize them roughly, as shown in the diagram, based on the extracted information, extraction approaches, and the language used in the documents being extracted.

In this section, we provide an overview of the current state of findings extraction from radiology reports. This includes an examination of existing methods, their associated challenges and limitations, as well as an exploration of the potential benefits achievable through the adoption of innovative frameworks and techniques. Figure 4 shows the schematic view of LMS for information extraction in medical imaging. By exploring these advancements, we aim to provide valuable insights into using language models to improve clinical decision-making in radiology.

In their research, Smit *et al* (2020) presented CheXbert, a BERT-based method for radiology report labeling in x-ray modalities, utilizing the CheXpert and MIMIC-CXR datasets. They employed a modified BERT architecture with 14 linear heads to address diverse medical observations, trained the model using cross-entropy loss and Adam optimization with fine-tuning of all BERT layers, and evaluated its performance through weighted-F1 metrics across positive, negative, and uncertainty extraction tasks, providing 95% confidence intervals through bootstrapping. The results showcased CheXbert’s superiority, achieving an F1 score of 0.798, surpassing previous models, including the state-of-the-art CheXpert labeler, and matching the performance of a board-certified radiologist, marking a significant advancement in radiology report labeling by amalgamating the scalability of rule-based systems with expert annotation quality.

Furthermore, domain adaptation can significantly enhance the performance of language models when extracting findings. In this study by Dhanaliwala *et al* (2023), the research demonstrated that the domain-adapted RadLing system surpasses the general-purpose GPT-4 in extracting common data elements (CDEs) from x-ray and CT reports, emphasizing the importance of specialized language models for medical information extraction. RadLing’s two-phase approach utilizes a Sentence Transformer, addressing data imbalance and achieving high sensitivity (94% versus 70%) but slightly lower precision (96% versus 99%) compared to GPT-4. Importantly, RadLing offers practical benefits such as local deployment and cost-efficiency, making it a promising tool for structured information extraction in radiology.

In some cases, extracting intricate spatial details from the radiology report presents greater challenges. The research employs two methods for extracting spatial triggers from radiology reports. The baseline method used a BERTBASE model, fine-tuned on the annotated corpus, treating it as a sequence labeling task. The proposed hybrid method included candidate trigger generation through exact matching and domain-specific constraints, followed by a BERT-based classification model to determine correctness. Both methods used BERTBASE, pre-trained on MIMIC-III clinical notes, and the evaluation includes rule-based approaches. While the sequence labeling method had high precision but low recall, exact matching improved recall significantly but resulted in many false positives. Applying constraints to exact matched triggers enhanced precision slightly. However, the hybrid approach, which combined domain-inspired constraints with a BERT-based classifier, achieved both balanced precision and recall. It exhibited an impressive 24-point improvement in F1 score compared to standard BERT sequence labeling, with an average accuracy of 88.7% over 10-fold cross-validation.

The pursuit of detailed information extraction leads to exploring linguistic diversity, specifically the challenges in processing German in medical texts. In a recent study by Jantscher *et al* (2023), they developed an information extraction framework for improved medical text comprehension in low-resource languages like German. Their approach, using a transformer-based language model for named entity recognition (NER) and relation extraction (RE) in German radiological reports, showed enhanced performance in medical predictive studies. By incorporating active learning and strategic data sampling techniques, they reduced the effort required for data labeling. Additionally, they introduced a comprehensive annotation scheme that integrated medical ontologies. The results demonstrated significant performance gains, particularly in NER and RE tasks, when transitioning from random to strategic sampling. Furthermore, adapting models across different clinical domains and anatomical regions proved highly effective, underscoring the model’s versatility.

This exploration of linguistic diversity sets the stage for examining German BERT models, showcasing tailored solutions for language-specific radiological data. A study by Dada *et al* (2024) demonstrated the effectiveness of German BERT models pre-trained on radiological data for extracting information from loosely structured radiology reports. This has significant implications for clinical workflows and patient care. Using two datasets for training and two BERT models, they achieved noteworthy results. RadBERT excelled in pre-training, with an HST accuracy of 48.05% and a 5HST accuracy of 66.46%. During fine-tuning, G-BERT+Rad+Flex had the highest F1-score (83.97%), and GM-BERT+Rad+Flex achieved the highest EM (Exact match) score (71.81%) in the RCQA task. Additionally, GM-BERT+Rad+Flex showed the highest accuracy for unanswerable questions, and models performed slightly better on CT reports. Strong generalization capabilities were observed across various categories.

Besides German, some researchers have also attempted work in other languages, such as Spanish. In a past study by López-Úbeda *et al* (2021), pre-trained BERT-based models were introduced for automating the extraction of biomedical entities from Spanish radiology reports, addressing a crucial need in Spanish medical text processing. Using three approaches—BERT multi-class entity, BERT binary class entity, and a BERT Pipeline—the study evaluated these methods on a dataset of 513 annotated ultrasonography reports, recognizing seven entity types and three radiological concept hedging cues. Notably, the BERT Binary class entity approach outperformed others, achieving an 86.07% precision and a significant 14% F1 improvement compared to the BERT multi-class entity method, reaching 67.96%. This method particularly excelled in recognizing specific entities, including negated entities, underscoring its effectiveness in Spanish radiology report entity detection.

The summary of publications related to finding extraction is presented in table 2. Table 3 summarizes the specific limitations and future perspectives of the reviewed papers, along with the common limitations and future perspectives discussed in this section.

**Table 2.** Overview of LMs for extracting findings from medical imaging documents. The asterisks (*) indicate terms that are either not present in the original paper or do not apply in this context.

References	ROI	Modality	Dataset	Model name	Base Model/structure
Datta and Roberts (2020)	Brain, Chest	x-ray, MRI	MIMIC III	*	BERT
Smit *et al* (2020)	Chest	x-ray	CheXpert, MIMIC-CXR	ChestXbert	BERT
Dhanaliwala *et al* (2023)	Chest	x-ray, CT	Institutional	RadLing	ELECTRA, BERT
Jantscher *et al* (2023)	Head, Pediatric	CT, MRI, x-ray	Institutional	*	BERT
Dada *et al* (2024)	*	CT, MRI	Institutional, DocCheck Flexikon	G-BERT, GM-BERT	BERT
Moon *et al* (2022)	Chest	x-ray	MIMIC-CXR, Open-I	MedViLL	ResNet-50, BERT
Singh *et al* (2022)	Cardiac structure	MRI	EWOC	*	BERT
López-Úbeda *et al* (2021)	*	*	SpRadIE	BETO	BERT

**Table 3.** Comparative assessment of limitations and future perspectives for research in finding extraction.

References	Specific limitation	Specific future perspective
Datta and Roberts (2020)	Rare phrases, common english terms, verb phrases	Generalization, rule expansion, POS information
Smit *et al* (2020)	Dependency on existing labeler, token limitation, limited to 14 observations, single radiologist ground truth, generalization across institutions	Reducing dependency on labelers, handling longer reports, expanding to rarer conditions, multiple radiologists for ground truth, cross-institutional evaluation
Dhanaliwala *et al* (2023)	Limited generalizability, evaluation scope, small cohort, data and model version, benchmarking method	Expanded scope, incorporating real-world complexity, model and data updates, comparative evaluation, integration into clinical workflows
Jantscher *et al* (2023)	Single-center study, limited open data sources, specific clinical domain	Evaluation across clinical sites, open language models, expansion to different clinical domains
Dada *et al* (2024)	Single text span answers, language restriction, interpretability	Multiple span models, language extension, interpretability research
Singh *et al* (2022)	Small test set, postprocessing requirements, portability, clinical implementation challenges, quality control	Larger test set, reduced postprocessing, enhanced portability, clinical implementation strategies, continuous monitoring
López-Úbeda *et al* (2021)	Entity recognition variability, dependency on pre-trained models	Error analysis, linguistic features, ontologies

### 3.2. Image/video captioning

**Figure 5.**
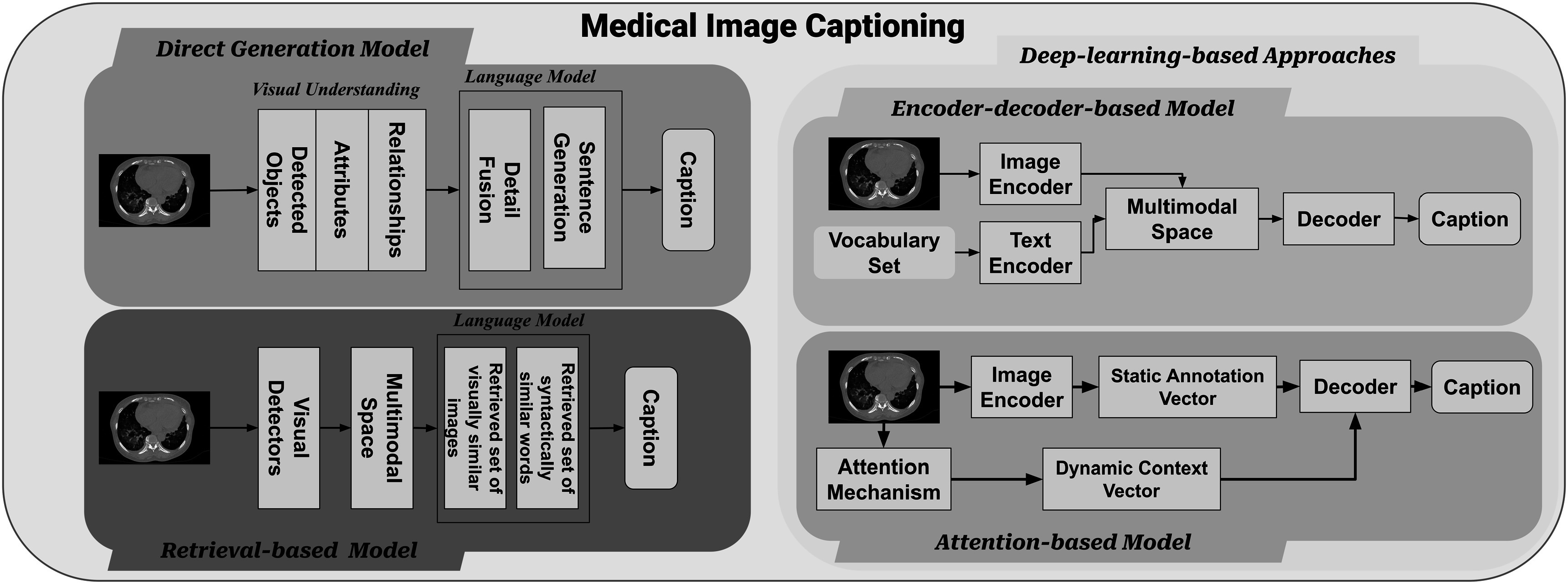
This diagram illustrates the process of medical image captioning using language models. Based on different captioning methods, it can be broadly categorized into direct generation approaches, retrieval-based approaches, and deep learning-based approaches. Among the deep learning approaches, there is further classification based on the involvement of an attention mechanism, leading to encoder–decoder-based approaches and attention-based approaches. Most medical image captioning methods primarily focus on encoder–decoder approaches.

Generating accurate and reliable automatic report generation systems for medical images poses several challenges, including analyzing limited medical images using machine learning approaches and generating informative captions for images involving multiple organs. For instance, captioning fetal ultrasound images presents a unique set of challenges due to their inherent complexity and significant variability. Fetal ultrasound images are often noisy, low-resolution, and subject to great variation depending on factors such as fetal position, gestational age, and imaging plane. Additionally, there is a lack of large-scale annotated datasets for this task. To address these challenges, researchers have proposed novice approaches that integrate visual and textual information to generate informative captions for images and videos. In figure 5, the diagram illustrates the different processes of medical image captioning using language models.

In their research, Nicolson *et al* (2021) applied a sequence-to-sequence model, incorporating pre-trained ViT and PubMedBERT checkpoints, to the task of medical image captioning. The model followed a vision-language architecture, with a ViT-based encoder for processing medical images and a decoder utilizing the pre-trained PubMedBERT checkpoint for generating captions. The encoder segmented images into non-overlapping patches, adds position embeddings, and aggregates representations. The decoder incorporateed token embeddings, position embeddings, and segment embeddings. Interestingly, the best-performing configuration, ‘vit2mrt-0.1.1_5_e131,’ eschewed various additional steps. However, a notable gap between validation and test scores raises questions of potential overfitting or dataset disparities, and the study highlighted the complexity of optimizing models for medical image captioning, with no universal solution.

Building on the foundation of pre-trained models, another study introduced a specialized approach for CT image captioning, combining advanced neural network architectures. In this paper, Kim *et al* (2023b) introduced a novel CT image captioning model that combined 3D-CNN encoders (ResNet-50, EfficientNet-B5, DenseNet-201, and ConvNeXt-S) with a distilGPT2 decoder to address continuous CT scan sequence captioning. Their approach employed cross-attention, teacher forcing, and a penalty-applied loss function, particularly enhancing intracranial hemorrhage detection. The EfficientNet-B5 model, trained with penalty-applied loss functions, achieved the highest scores in metrics like BLEU, METEOR, and ROUGE-L, outperforming models using standard loss functions. This highlights the efficacy of penalty-based loss functions in capturing critical medical information.

Transitioning from specific imaging modalities to a more general approach, the next research utilized a comprehensive encoder-to-decoder model, aiming to address the diversity of medical imaging. Nicolson *et al* (2023) proposed a concise encoder–decoder model for medical image captioning, optimized with self-critical sequence training (SCST) (Rennie *et al* 2017) and focusing on BERTScore. This approach addressed the challenge of generating coherent captions for diverse medical images, with relevance for clinical documentation and multimodal analysis. The model employed CvT-21 as the image encoder and DistilGPT2 as the caption decoder, trained using a two-stage process with teacher forcing (TF) (Toomarian and Barhen 1992) and SCST. The model achieved top-ranking results in BERTScore and performed well in other metrics like CLIPScore, ROUGE, and CIDEr, despite lower ranks in METEOR and BLEU due to SCST optimization with BERTScore.

Further enhancing the capabilities of medical image captioning, the subsequent study adopted the BLIP framework, demonstrating its efficacy in a medical context. Wang and Li (2022) use the BLIP framework for medical image captioning, improving verbal fluency and semantic interpretation in a medical context. They employed the multimodal mixture of encoder–decoder (MED) and dual pre-training strategies. Pre-training from scratch on the ImageCLEFmedical dataset had limited success, but secondary pre-training with the original BLIP model achieved a notable BLEU score of 0.2344 when fine-tuned on the validation set, demonstrating its effectiveness in medical image captioning.

Expanding upon the use of the BLIP framework, the following research explored its application in a more focused area, introducing a novel classification pipeline. Ding *et al* (2023) introduced a mitosis classification pipeline using large vision-language models (BLIP) that framed the problem as both image captioning and visual question answering, incorporating metadata for context. BLIP outperformed CLIP models, with significantly higher F1 scores and AUC on MIDOG22 dataset. Adding metadata enhances BLIP’s performance, while CLIP did not surpass vision-only baselines, highlighting the advantage of multimodal vision and language integration for mitosis detection in histopathology images.

Moving from specific applications to a more integrative approach, MSMedCap (Wang *et al* 2023a) presented a model that combined dual encoders with large language models, demonstrating a unique methodology in image captioning. Guided by SAM, the model captured diverse information in medical images. MSMedCap integrated dual image encoders (fCLIP and fSAM) to capture general and fine-grained features, improving caption quality over baseline models BLIP2 and SAM-BLIP2. Mixed semantic pre-training in MSMedCap effectively combined CLIP’s general knowledge and SAM’s fine-grained understanding, showcasing its strength in generating high-quality medical image captions.

During the captioning process, there may be instances where interaction with human users is desirable. Zheng and Yu (2023) contributed by developing an interactive medical image captioning framework that effectively involves human users, integrated evidence-based uncertainty estimation, and provided a theoretical basis for uncertainty integration, thus improving the accuracy of image captions in a data-efficient manner. Their novel approach combined evidential learning with interactive machine learning for medical image captioning, progressing through stages from keyword prediction to caption generation, with user feedback informing refinement. Evaluations on two medical image datasets demonstrated the model’s capacity for efficient learning with limited labeled data, achieving superior performance compared to other models across various metrics on the PEIR and MIMIC-CXR datasets.

The summary of publications related to medical image/video captioning is presented in table 4. Table 5 summarizes the specific limitations and future perspectives of the reviewed papers, along with the common limitations and future perspectives discussed in this section.

**Table 4.** Overview of LMs for captioning medical images. The asterisks (*) indicate terms that are either not present in the original paper or do not apply in this context.

References	ROI	Modality	Dataset	Model name	Vision model	Language model
Nicolson *et al* (2023)	*	x-ray, Ultrasound, CT, MRI	Radiology objects in context (ROCO)	CvT2DistilGPT2	CvT-21	DistilGPT2
Nicolson *et al* (2021)	*	CT, Ultrasoud, x-ray, Fluroscopy, PET, Mammography, MRI, Angiography	ROCO, ImageCLEFmed Caption 2021	*	ViT	PubMedBERT
Kim *et al* (2023b)	Brain	CT	Institutional	*	ResNet-50, EfficientNet-B5, DenseNet-201, and ConvNeXt-S	DistilGPT2
Zheng and Yu (2023)	*	*	IU-Xray	*	CDGPT (Conditioned Densely-connected Graph Transformer)	AlignTrans (Alignment Transformer)
Wang and Li (2022)	*	*	ImageCLEFmedical Caption 2022	BLIP	Vision Transformer (ViT-B)	BERT
Ding *et al* (2023)	*	Histopathology	MIDOG 2022	CLIP, BLIP	Vision Transformer (ViT-B)	BERT
Wang *et al* (2023a)	*	*	ImageCLEFmedical Caption 2022	BLIP	Vision Transformer (ViT-B)	BERT
Zhou *et al* (2023)		Ultrasound, CT, x-ray, MRI	ROCO	BLIP-2	ViT-g/14	OPT2.7B

**Table 5.** Comparative assessment of limitations and future perspectives for research in image and video captioning.

References	Specific limitation	Specific future perspectives
Nicolson *et al* (2023)	Discrepancy between validation and test scores; sensitivity of SCST; noise in PubMed central datasets	Clinical relevance metrics; ethical considerations; learning rate optimization
Kim *et al* (2023b), Wang *et al* (2023a)	Spatial information handling; performance on normal CT scans	Specialized language models; advanced spatial analysis; contextual information
Wang *et al* (2023a)	Suboptimal results with BLIP2; confusion due to irrelevant training details	Incorporating medical knowledge; specialized evaluation metrics

### 3.3. Diagnosis interpretability

Medical image analysis and diagnosis have long been challenging due to the need for more interpretability of deep neural networks, limited annotated data, and complex biomarker information. These challenges have inspired researchers to develop innovative AI-based methods that can automate diagnostic reasoning, provide interpretable predictions, and answer medical questions based on raw images.

Monajatipoor *et al* (2022) made a significant contribution with BERTHop, a specialized vision-and-language model tailored for medical applications. BERTHop specifically addressed the challenge of interpretability in medical imaging by combining PixelHop++ (Chen *et al* 2020b) and VisualBERT (Li *et al* 2019). This innovative approach allowed the model to effectively capture connections between clinical notes and chest x-ray images, leading to improved interpretability in disease diagnosis. On the OpenI dataset, BERTHop achieved remarkable performance with an average AUC of 98.23% when diagnosing thoracic diseases from CXR images and radiology reports, outperforming existing models in the majority of disease diagnoses. Additionally, the study highlighted the benefits of initializing with in-domain text data for further improving model performance.

Building on the interpretability enhanced by BERTHop, the next advancement, ChatCAD (Wang *et al* 2023b), integrated large language models (LLMs) with computer-aided diagnosis networks, further bridging AI and clinical practice. This framework enhanced diagnosis interpretability by summarizing and reorganizing CAD network outputs into natural language text, creating user-friendly diagnostic reports. ChatCAD’s approach involved translating CAD model results into natural language, utilizing LLMs for summarization and diagnosis, and incorporating patient interaction. Evaluation on medical datasets demonstrated ChatCAD’s strong performance, outperforming existing methods for key disease observations on the MIMIC-CXR dataset in terms of precision, recall, and F1-score. ChatCAD excelled in providing informative and reliable medical reports, enhancing diagnostic capabilities and report trustworthiness.

Expanding from specific AI-language model integrations, the focus shifts to a unified framework that leverages natural language concepts for a more robust and interpretable medical image classification (Yan *et al* 2023a). This approach enhanced diagnostic accuracy, trustworthiness, and interpretability by associating visual features with medical concepts. The framework outperformed various baselines in datasets with confounding factors, demonstrating its robustness. It also provided concept scores, classification layer weights, and instance-level predictions for improved interpretability in medical image classification.

Narrowing the scope from a general framework to a specialized application, the next study introduced BCCX-DM (Chen *et al* 2021), specifically targeting breast cancer diagnosis and enhancing accuracy through a novel XAI model. The model included three components: structuring mammography reports, transforming TabNet’s structure with causal reasoning, and using the Depth-First-Search algorithm and gated neural networks for non-Euclidean data. In experiments, Causal-TabNet achieved better accuracy (0.7117) compared to TabNet (0.6825) when processing 21 features in classifying benign and malignant breast tumors. The model’s interpretability excelled by identifying critical diagnostic factors, such as tumor boundaries and lymph nodes, aligning with clinical significance.

Further advancing the connection between medical images and textual data, the MONET model (Kim *et al* 2023a) represented a significant leap in AI transparency, particularly in the field of dermatology. This model enhanced AI transparency by connecting medical images with text and generating dense concept annotations, particularly in dermatology. MONET, when combined with a Concept Bottleneck Model (MONET+CBM), consistently outperformed other methods in malignancy and melanoma prediction tasks, with mean AUROC values of 0.805 for malignancy and 0.892 for melanoma prediction. These results were statistically significant (*p* < 0.001) and demonstrate MONET’s superiority. Also contributed in dermatology, Patrício *et al* (2023) improved language models for skin lesion diagnosis, enhancing interpretability and performance. Their method achieved consistent performance gains over CLIP variations across three datasets. It also provided concept-based explanations for melanoma diagnosis.

The summary of publications related to interpretability improvement is presented in table 6. Table 7 summarizes the specific limitations and future perspectives of the reviewed papers, along with the common limitations and future perspectives discussed in this section.

**Table 6.** Overview of LMs for improving diagnosis interpretability. The asterisks (*) indicate terms that are either not present in the original paper or do not apply in this context.

References	ROI	Modality	Dataset	Model name	Base model/structure
Chen *et al* (2021)	Breast	Mammography	Institutional	BCCX-DM	TabNet, causal bayesian networks, gated neural networks (GNN)
Monajatipoor *et al* (2022)	Chest	x-ray	Open-I	BERTHop	PixelHop++, BlueBERT
Wang *et al* (2023b)	Chest	x-ray	MIMIC-CXR, CheXpert	ChatCAD	GPT-3, CAD
Kim *et al* (2023a)	Chest	x-ray	Fitzpatrick 17k, DDI	MONET	ResNet, CLIP
Yan *et al* (2023a)	Chest	x-rays	NIH-CXR, Covid-QU, Pneumonia, Open-I	—	GPT4, BioVil
Patrício *et al* (2023)	Skin	Dermascope	PH2, Derm7pt, ISIC2018	CLIP	BERT

**Table 7.** Comparative assessment of limitations and future perspectives for research in diagnosis interpretability.

References	Specific limitation	Specific future perspective
Monajatipoor *et al* (2022)	Data annotation; visual encoder choices; evaluation scope	Larger annotated datasets; visual encoder flexibility; diverse medical imaging tasks
Wang *et al* (2023b)	Non-human-like language generation; limited prompts; impact of LLM Size	Language model refinement; exploration of more complex information; investigation of vision classifiers; quantitative analysis of prompt design; engagement with clinical professionals
Yan *et al* (2023a)	Potential biases; accuracy of concept scores	Improved language models; robustness enhancements; reducing model dependency; biased data mitigation
Patrício *et al* (2023)	Interpretable concept	Expanding to other imaging modalities; enhanced interpretability

### 3.4. Report classification

In this session, we will be discussing the challenges faced in report classification for medical imaging. The manual labeling process of radiology reports for computer vision applications is time-consuming and labor-intensive, creating a bottleneck in model development. Extracting clinical information from these reports is also a challenge, limiting the efficiency and accuracy of clinical decision-making. The goal of this research area is to automate this process, improving efficiency and accuracy in the medical field.

Bressem *et al* (2020) successfully applied BERT models to accurately classify chest radiographic reports, achieving high accuracy in identifying important findings. They utilized four BERT-based models, including GER-BERT, MULTI-BERT, FS-BERT, and RAD-BERT, trained and evaluated on a vast dataset of radiological reports. RAD-BERT emerged as the top-performing model with a pooled AUC of 0.98 and an AUPRC of 0.93 when fine-tuned on a 4000-text training dataset. However, performance varied by finding, with pneumothorax being challenging due to class imbalance. RAD-BERT also excelled in classifying CT reports, showcasing its promise in radiology report classification.

Expanding from this foundational use of BERT models, the next study introduced FlexR (Keicher *et al* 2022), a method that elevated the standard of automated reporting in chest x-rays through few-shot learning techniques. This addressed the need for standardized and automated reporting in radiology. FlexR leveraged self-supervised pretraining to extract clinical findings from structured report templates, maps them into a joint language-image embedding space, and fine-tunes a classifier. Using the MIMIC-CXR-JPG dataset, it excelled in tasks like cardiomegaly severity assessment and pathology localization with limited annotated data, achieving significant AUC improvements over baselines in both 1-shot and 5-shot learning scenarios.

Moving beyond chest x-rays to a broader range of imaging modalities, the following study focuses on automating the labeling of MRI radiology reports with the innovative ALARM model (Wood *et al* 2020). ALARM modified and fine-tunes the BioBERT language model, outperforming expert neurologists and stroke physicians in classification tasks. It achieved remarkable accuracy, sensitivity, and specificity in binary classification and granular abnormality categorization, showcasing its potential in radiology report labeling.

Delving deeper into the specialization of neural networks for imaging, the subsequent research (Wood *et al* 2022) shifted focus to the efficient deep learning approach in automating head MRI dataset labeling. Their sophisticated neural network model, trained on a large-scale dataset with meticulous labeling, demonstrated high efficacy in classifying head MRI examinations based on radiology reports, achieving impressive results with an AUC-ROC over 0.95 for all categories against reference-standard report labels. While there was a slight performance drop when tested against reference-standard image labels, the model’s robustness in automated MRI dataset labeling is evident, particularly in neuroradiology reports.

Finally, Zhang *et al* (2023b) introduced connect image and text embeddings (CITE), an innovative framework that merged biomedical text with foundation models to enhance pathological image classification. This model combined a pre-trained vision encoder and a biomedical language model to align image and class name embeddings, significantly improving classification accuracy. In their evaluation on the PatchGastric dataset, focusing on gastric adenocarcinoma subtypes, CITE consistently outperformed all baseline models by a significant margin, showcasing its effectiveness in handling limited medical data and enhancing pathological image diagnosis.

The summary of publications related to report classification presented in table 8. Table 9 summarizes the specific limitations and future perspectives of the reviewed papers, along with the common limitations and future perspectives discussed in this section.

**Table 8.** Overview of LMs for report classification. The asterisks (*) indicate terms that are either not present in the original paper or do not apply in this context.

References	ROI	Modality	Dataset	Model Name	Base model/structure
Wood *et al* (2020)	Head	MRI	Institutional	ALARM	BIOBERT, custom attention mechanism
Wood *et al* (2022)	Head	MRI	Institutional	BioBERT	BERT
Huemann *et al* (2023b)	*	PET, CT	Institutional	BERT, BioClinicalBERT, RadBERT, and RoBERTa	BERT
Keicher *et al* (2022)	Chest	x-ray	MIMIC-CXR-JPG v2.0.0	FlexR	CLIP, language embedding, fine-tuning classifier
Bressem *et al* (2020)	Chest	x-ray, CT	Institutional	GER-BERT, MULTI-BERT, FS-BERT, and RAD-BERT	BERT
Zhang *et al* (2023b)	*	Histopathology	PatchGastric	CITE	CLIP, ImageNet-21k, CLIP textual encoder, BioBERT (BB), and BioLinkBERT (BLB)​

**Table 9.** Comparative assessment of limitations and future perspectives for research in report classification.

References	Specific limitation	Specific future perspective
Wood *et al* (2020)	Granular classifiers as work in progress; semi-supervised image labeling for unlabeled data	Expanding training dataset; utilizing web-based annotation tools for refined labeling
Wood *et al* (2022)	Less accurate granular label assignment; mislabeling in neuroradiology report classifiers	Investigating classifier generalizability; refining model to reduce label noise
Huemann *et al* (2023b)	Data source diversity; label variability; single prediction task focus; limited human versus ai comparison; token limitation in language models; image processing methodology	Expanding task range; utilizing diverse pretraining data; conducting multi-reader studies; addressing token limitation; incorporating full-text reports; evaluating in diverse institutions
Keicher *et al* (2022)	Data availability for self-supervised pretraining; disease localization challenges	Refining pretraining; explicit modeling of label dependencies; developing standardized reporting templates; implementing negative prompts strategy; enhancing fine-grained clinical findings; improving data accessibility
Bressem *et al* (2020)	Model size limitation; language specificity; bias in labeling short reports	Integration of single-finding reports; utilizing larger BERT models; developing multilingual models; handling longer texts efficiently; generalization of RAD-BERT
Zhang *et al* (2023b)	Data-limited challenges; dependency on medical domain knowledge	Use of synthetic pathological images; foundation training on multi-modal medical images

### 3.5. Report generation

This section is dedicated to exploring the advancements in language models for generating medical reports. which has become an increasingly important task in the field of computer-aided diagnosis. The challenges of generating accurate and readable medical reports from various types of medical images have motivated researchers to explore new methods for automatic report generation. The authors of the studies discussed here were motivated by the task of generating diagnostic reports with interpretability for various medical imaging modalities, including computed tomography (CT) volumes, skin pathologies, ultrasound images, and brain CT imaging. The progress in creating precise and efficient language models specifically designed for medical report generation has the potential to enhance the effectiveness of clinical workflows, mitigate diagnostic errors, and provide valuable support to healthcare professionals in delivering timely and precise diagnoses.

Leonardi, Portinale *et al* provided a vital methodology centered on reusing pre-trained language models, offering the potential to automate radiological report generation from x-ray images in healthcare (Leonardi *et al* 2023). The model was based on a decoder-only transformer architecture, customized for the generation of textual reports from radiological images. It integrated input embedding, self-attention mechanisms, and an output layer. To adopt a multimodal approach, an image encoder was incorporated. During the training process, teacher forcing with cross entropy loss was utilized. The evaluation tasks focused on generating textual descriptions for medical images, making use of datasets such as MIMIC-CXR. This adaptation significantly improved the model’s capacity to process visual data and generate precise textual reports. The most remarkable combination, ViT + GPT-2 with Beam Search, attained the highest performance, achieving a precision of 0.79, recall of 0.76, and an impressive F1-score of 0.78. These outcomes outperformed alternative model architectures and decoding methods, highlighting the importance of capturing semantic and contextual information to generate high-quality text that aligns closely with human judgment.

Expanding upon the foundation of pre-trained models, Clinical-BERT (Yan and Pei 2022) emerged as a tailored vision-language pre-training model, enhancing domain-specific knowledge integration in radiology diagnosis and report generation. Clinical-BERT incorporated domain-specific pre-training tasks, including Clinical Diagnosis, Masked MeSH Modeling, and Image-MeSH Matching, alongside general pre-training tasks like masked language modeling. Radiographic visual features and report text were combined to create token embeddings. The model was pre-trained on the MIMIC-CXR dataset, utilizing BERT-base as the base model and DenseNet121 as the visual feature extractor. Fine-tuning for specific tasks involved variations in data ratios, tokenizations, and loss functions. Clinical-BERT demonstrated exceptional performance in Radiograph Report Generation, securing top rankings in NLG metrics on IU x-ray and substantially improving clinical efficacy metrics, with precision, recall, and F1 scores showing an average gain of 8.6% over a competing model. Ablation studies underscored the effectiveness of domain-specific pre-training tasks.

Furthering the advancement in report generation, the next study focused on cross-institutional solutions, addressing the challenge of model generalization in diverse healthcare environments. The ChatRadio-Valuer model (Zhong *et al* 2023) combined computer vision and advanced language models to extract clinical information from radiology images and generate coherent reports. It trained on a diverse dataset from multiple institutions, demonstrating robust performance and high clinical relevance. Evaluation metrics showed its superior performance compared to 15 other models, particularly excelling in Recall-1, Recall-2, and ROUGUE-L scores, especially for datasets from Institutions 2, 5, and 6. Within Institution 1, it adapted well across various systems, making it a promising tool for enhancing diagnostics and clinical decision-making in diverse medical settings.

Building on these advancements, MedEPT (Li 2023) proposed a method enhancing data and parameter efficiency, reducing the reliance on extensive human-annotated data. MedEPT featured three key components: a CLIP-inspired feature extractor with separate image and text encoders and projection heads, a lightweight transformer-based mapping network, and a parameter-efficient training approach. It used soft prompts for text generation and was trained on various medical datasets, including MIMIC-CXR, RSNA Pneumonia, and COVID. MedEPT consistently outperformed existing models across metrics like BLEU-1, BLEU-4, Rouge, and CIDEr, even requiring less training time and fewer parameters. The study emphasized the significance of diverse text generation techniques, language model choice, and parameter-efficient tuning in optimizing performance.

RGRG (Tanida *et al* 2023), which innovatively integrated anatomical region detection with interactive report generation, enhanced clinical relevance and report completeness. The RGRG method encompassed radiology report generation, anatomy-based sentence generation, and selection-based sentence generation, mirroring a radiologist’s workflow. It comprised four main modules: object detection, region selection, abnormality classification, and a transformer-based language model. The model underwent training in three stages, beginning with the object detector, and evaluation encompassed standard language generation and clinical efficacy metrics. In radiology report generation, it outperformed previous models, achieving a new state-of-the-art in METEOR and excelling in clinically relevant CE metrics. For anatomy-based sentence generation, it produced pertinent anatomy-related sentences with a notable anatomy-sensitivity-ratio. In selection-based sentence generation, the model demonstrated robustness to deviations in bounding boxes.

Advancing the integration of clinical knowledge, the KiUT (Huang *et al* 2023) represented a significant leap in report generation, incorporating a novel architecture and a symptom graph for more accurate and clinically informed reports. KiUT, a comprehensive solution for generating informative radiology reports from 2D images, consisting of three core components: cross-modal U-transformer, injected knowledge distiller, and region relationship encoder. The U-Transformer enhanced feature aggregation through multi-modal interaction, while the injected knowledge distiller aligned the model with medical expertise by combining visual, contextual, and clinical knowledge. The region relationship encoder captured image region relationships for contextually relevant reports. On the MIMIC-CXR dataset, KiUT outperformed competing models, achieving higher NLG metrics with BLEU-1 scores of 0.393 and ROUGE-L scores of 0.285, demonstrating its proficiency in generating coherent and clinically relevant reports. Additionally, KiUT excelled in describing clinical abnormalities on CheXpert labels for 14 disease categories, and it surpassed most models on the IU-Xray dataset.

Setting a new standard in both clinical efficacy and explainability in medical report generation, transformer-based semantic query (TranSQ) (Kong *et al* 2022) introduced a novel candidate set prediction and selection method. This model was comprised of three components: the visual extractor, semantic encoder, and report generator. It used ViT to convert medical images into visual features, processed by semantic queries to create semantic features. The report generator produced sentence candidates through retrieval and predicted selection probabilities with a multi-label classifier. TranSQ achieved state-of-the-art results, including a BLEU score of 0.608 and an F1 score of 0.642 on the IU x-ray dataset, and demonstrated impressive clinical efficacy on the MIMIC-CXR dataset with a precision score of 0.482, recall score of 0.563, and an F1-score of 0.519, surpassing the state-of-the-art model KGAE. Remarkably, TranSQ achieved these improvements without introducing abnormal terms and maintained its state-of-the-art performance even with random-ordered sentences.

The summary of publications related to report generation is presented in table 10. Table 11 summarizes the specific limitations and future perspectives of the reviewed papers, along with the common limitations and future perspectives discussed in this section.

**Table 10.** Overview of LMs for report generation. The asterisks (*) indicate terms that are either not present in the original paper or do not apply in this context.

References	ROI	Modality	Dataset	Model name	Vision model	Language model
Zhong *et al* (2023)	Chest, Abdomen, musculoskeletal system, head, maxillofacial and neck	CT, MRI	Institutional (Six Chinese Hospital)	ChatRadio-Valuer	*	Llama2
Yan and Pei (2022)	Chest	x-ray	MIMIC-CXR, IU x-ray, COV-CTR, NIH ChestX-ray14	Clinical-BERT	DenseNet121	BERT-base
Leonardi *et al* (2023)	Chest	x-ray	MIMIC-CXR	*	ViT, CheXNet	Transformer
Li (2023)	Chest	x-ray	MIMIC-CXR, RSNA Pneumonia, COVID, IU Chest x-ray	*	CLIP	GPT-2, OPT-1.3B, OPT-2.7B
Tanida *et al* (2023)	Chest	x-ray	Chest ImaGenome v1.0.0	RGRG Method	ResNet-50, Faster R-CNN	Transformer
Huang *et al* (2023)	Chest	x-ray	IU-Xray, MIMIC-CXR	KiUT	ResNet101, U Transformer	BERT
Cao *et al* (2023)	Gastrointestinal tract, chest	Endoscope, x-ray	Gastrointestinal endoscope image dataset (GE), IU-CX, MIMIC-CXR	MMTN	DenseNet-121	BERT
Moon *et al* (2022)	Chest	x-ray	MIMIC-CXR, Open-I	MedViLL	CNN	BERT
Kong *et al* (2022)	Chest	x-ray	IU x-ray, MIMIC-CXR	TranSQ	ViT-B/32	MPNet

**Table 11.** Comparative assessment of limitations and future perspectives for research in report generation.

References	Specific limitation	Specific future perspective
Zhong *et al* (2023)	Focus only on chinese radiology reports; limited to ct and mri reports; inferior performance in certain report types; challenges in causal reasoning report generation; generalizability concerns in global contexts	Investigate performance with more diverse data; expand to other image modalities; improve performance in complex reports; enhance causal reasoning in reports; test and adapt in varied healthcare systems
Yan and Pei (2022)	MeSH word prediction accuracy	Incorporate organ localization; expand downstream tasks
Leonardi *et al* (2023)	Scarcity of data; localization errors; computational resource limitations; usability validation issues	Explore data augmentation techniques; improve localization accuracy; develop resource-efficient models; conduct usability assessments
Li (2023)	Limited data availability; model comparison constraints; caption diversity issues	Develop effective data augmentation methods; explore various advanced language models; enhance caption diversity and control
Tanida *et al* (2023)	Strong supervision reliance; focus on isolated chest x-rays; incomplete coverage of reference reports	Adapt for limited supervision; utilize sequential exam information; create hybrid systems for comprehensive sentence generation
Kong *et al* (2022)	Limited focus on abnormality term generation	Apply semantic features to a conditional linguistic decoder for enhanced sentence generation

### 3.6. Multimodal learning

Advancements in medical imaging technologies have led to an increase in the volume of image and text data generated in healthcare systems. To analyze these vast amounts of data and support medical decision-making, there is a growing interest in leveraging machine learning for automated image analysis and diagnosis. However, the effectiveness of these techniques is often limited by the challenges associated with bridging multimodal data, such as text and images.

**Figure 6.**
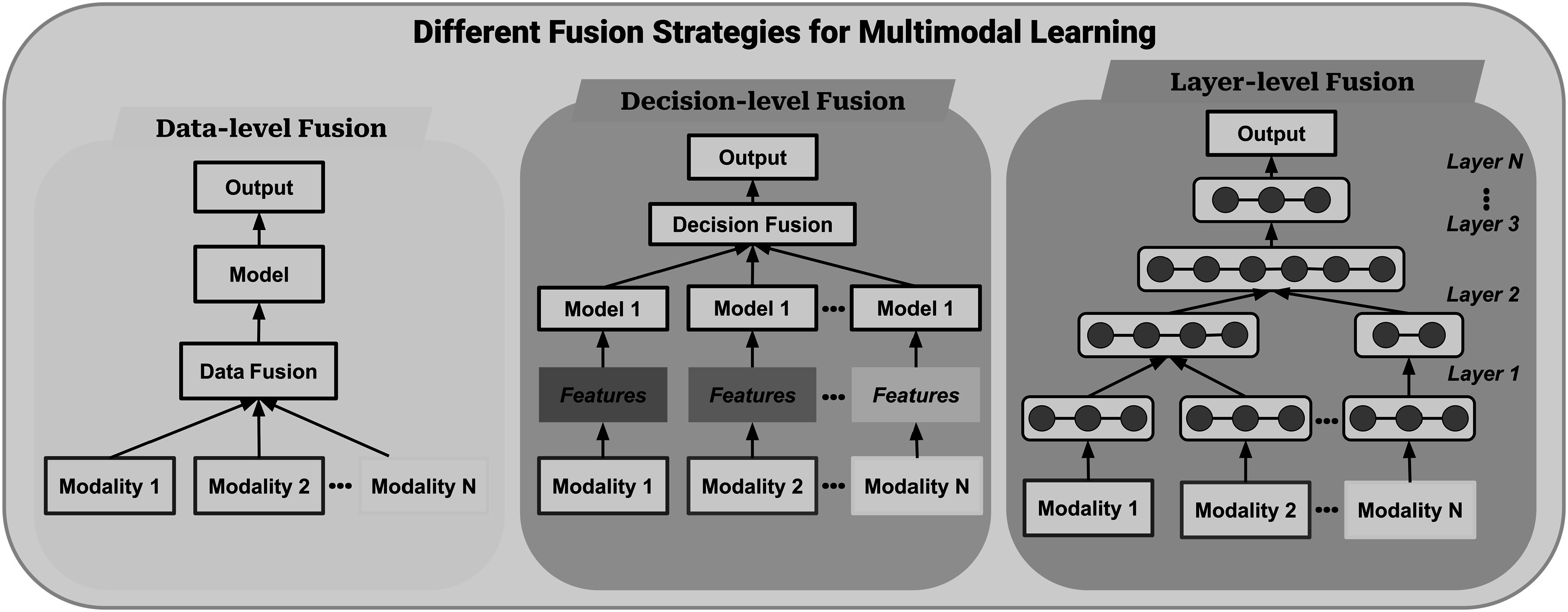
Illustration of various fusion strategies in multi-modal learning. This includes Data-level (Early) Fusion, where information from different modalities is combined at the input; Layer-level (Intermediate) Fusion, where fusion occurs at intermediate model layers; and Decision-level (Late) Fusion, where modalities are integrated at the final decision stage.

Multimodal learning has emerged as a promising approach for addressing these challenges in medical imaging. By leveraging both visual and textual information, multimodal learning techniques have the potential to improve diagnostic accuracy and enable more efficient analysis of medical imaging data. Figure 6 depicts the common strategies for fusing different modalities of data, primarily text and image data in the papers reviewed in our study.

Khare *et al* (2021) developed MMBERT, a Multimodal Medical BERT model, through self-supervised pretraining on a large medical image+caption dataset. This resulted in state-of-the-art visual question answering (VQA) performance for radiology images and improved interpretability via attention maps. MMBERT utilized a specialized Transformer encoder with self-attention, ResNet152 for image features, and BERT WordPiece tokenizer for text. Fine-tuning on medical VQA datasets achieved superior accuracy and BLEU scores compared to existing models, and qualitative analysis showed its ability to attend to relevant image regions and even surpass ground truth answers in some cases.

Expanding on the multimodal concept, ConTEXTual Net (Huemann *et al* 2023a) emerged, focusing specifically on enhancing pneumothorax segmentation in chest radiographs. By combining a U-Net-based vision encoder with the T5-Large language model and cross-attention modules, ConTEXTual Net achieved a Dice score of 0.716 for pneumothorax segmentation on the CANDID-PTX dataset, outperforming the baseline U-Net and performing comparably to the primary physician annotator. Vision augmentations significantly improved performance, while text augmentations did not. The choice of language model had minimal impact, and lower-level cross-attention integration improved results, with L4 module performing slightly better than L3.

Building further on multimodal interactions, GLoRIA (global-local representations for images using attenion mechanism) (Huang *et al* 2021) represented a significant step forward in jointly learning global and local representations of medical images. GLoRIA learns multimodal global and local representations of medical images through attention-weighted regions and paired reports. It employed attention mechanisms to emphasize image regions and reports during training, creating comprehensive representations. GLoRIA excelled in image-text retrieval, achieving high precision on the CheXpert dataset. In fine-tuned image classification, it performed remarkably well with AUROC scores of 88.1% (CheXpert) and 88.6% (RSNA) despite limited training data. In zero-shot image classification, it exhibited robust generalization with F1 scores of 0.67 (CheXpert) and 0.58 (RSNA). Furthermore, in segmentation tasks on the SIIM Pneumothorax dataset, GLoRIA attains competitive Dice scores, including a maximum of 0.634.

Leveraging textual annotations for image enhancement, the language meets vision transformer (LViT) model introduceed a novel approach in medical image segmentation, particularly addressing limited labeled data. LViT utilizes a double-U structure that combines a U-shaped CNN branch with a U-shaped Transformer branch, facilitating the integration of image and text information. A pixel-level attention module (PLAM) retains local image features, while an Exponential Pseudo-label Iteration mechanism and a language-vision (LV) Loss enable training with unlabeled images using direct text information. The tiny version of LViT, LViT-T, achieved significant improvements over the nnUNet model on the QaTa-COV19 dataset, enhancing the Dice score by 3.24% and the mean intersection over union (mIoU) score by 4.3%, even with just a quarter of the training labels, demonstrating its prowess in enhancing segmentation performance.

Besides segmentation, Huemann *et al* (2022) utilized NLP methods in clinical report interpretation, particularly in the context of lymphoma PET/CT imaging. Their multimodal model, combining language models like ROBERTA-Large pretrained on clinical reports and vision models processing PET/CT images, predicted 5-class visual Deauville scores (DS) with impressive accuracy. The ROBERTA language model achieved a 73.7% 5-class accuracy and a Cohen kappa (*κ*) of 0.81 using clinical reports alone, while the multimodal model, combining text and images, reached the highest accuracy of 74.5% with a *κ* of 0.82. Pretraining language models with masked language modeling (MLM) further improved performance, with ROBERTA reaching 77.4%.

Further exploring the synergy of vision and language, the development of multi-modal masked autoencoders (M3AE) (Chen *et al* 2022) highlights advancements in self-supervised learning paradigms. The M3AE architecture employs Transformer-based models for both vision and language encoders, which are combined using a multi-modal fusion module. In a self-supervised learning paradigm, the model masks and subsequently reconstructs portions of both images and text, facilitating joint learning of visual and textual representations. It demonstrated superior performance in Medical Visual Question Answering and medical image-text classification compared to previous uni-modal and multi-modal methods under the non-continued pre-training setting.

The summary of publications related to multimodal learning is presented in table 12. Table 13 summarizes the specific limitations and future perspectives of the reviewed papers, along with the common limitations and future perspectives discussed in this section.

**Table 12.** Overview of LMs for multimodal learning. The asterisks (*) indicate terms that are either not present in the original paper or do not apply in this context.

References	ROI	Modality	Dataset	Model name	Vision model	Language model
Huemann *et al* (2023a)	Chest	x-ray	CANDID-PTX	ConTEXTual Net	U-Net	T5-Large
Huang *et al* (2021)	Chest	x-ray	CheXpert, RSNA pneumonia detection challenge, SIIM-ACR pneumothorax segmentation, NIH ChestX-ray14	GLoRIA	CNN	Transformer
Li *et al* (2023)	*	CT, x-ray	MosMedData+, ESO-CT, QaTa-COV19	LViT	U-shaped CNN	U-shaped ViT (BERT-Embed)
Khare *et al* (2021)	*	*	VQA-Med 2019, VQA-RAD, ROCO	MMBERT	ResNet152	BERT
Huemann *et al* (2022)	Lymphoma	PET, CT	Institutional	—	ViT, EfficientNet B7	ROBERTA-Large, Bio ClinicalBERT, and BERT
Chen *et al* (2022)	*	x-ray, MRI, CT	ROCO, MedICaT	M^3^AE	ViT	BERT
Delbrouck *et al* (2022)	*	*	MIMIC-CXR, Indiana University x-ray collection, PadChest, CheXpert, VQA-Med 2021	ViLMedic	CNN	BioBERT

**Table 13.** Comparative assessment of limitations and future perspectives for research in multimodal learning.

References	Specific limitation	Specific future perspective
Huemann *et al* (2023a)	Single dataset; limited multimodal datasets; primary annotator bias; language as input requirement; comparison challenges	Diverse datasets; inter-observer variability; task expansion; reducing language dependency; AI-assisted clinical workflow
Huang *et al* (2021)	Focus on chest radiographs; dependence on report quality; designed for english-language reports	Expand to other modalities and regions; improve robustness to report quality; adapt for different languages
Li *et al* (2023d)	2D segmentation limitation; manual text annotation requirement	3D segmentation; automating text annotation generation
Khare *et al* (2021)	Small labeled datasets; model interpretability; incomplete consideration of expert diagnosis	Larger datasets; enhanced interpretability; clinical integration
Chen *et al* (2022)	Complexity and diversity handling in medical image-text data	Sophisticated techniques for diverse medical scenarios
Delbrouck *et al* (2022)	Visio-linguistic reasoning and understanding; overfitting and generalization; transparency in medical AI; NLG evaluation in medical AI	Enhanced interpretability in multimodal scenarios; refined training methodologies; improved documentation and accessibility; advanced evaluation metrics

### 3.7. Visual question answering

VQA in medical imaging combines image processing and natural language processing to enable machines to answer clinical questions associated with medical images. In the dermatology field, the increasing prevalence of skin diseases and the challenges faced by patients in accessing dermatologists, particularly in remote or underdeveloped areas, have inspired researchers to explore innovative approaches to accurately diagnose skin diseases. Existing attempts at image classification for skin disease diagnosis have been limited by small datasets with few classes, highlighting the need for more comprehensive approaches. Meanwhile, the critical role that medical images play in clinical and healthcare domains has emphasized the need for solutions that can accurately interpret these images, even for experts. VQA models for medical imagery can help to improve the accuracy of diagnosis and treatment by enabling machines to understand and interpret medical images. In this session, we examine recent advancements in VQA models for medical imaging and discuss their potential impact on diagnosis and treatment in the medical field.

A medical VQA system that emphasized capturing essential visual representations was introduced, which is crucial for addressing challenges in the medical domain, including limited training data and distinguishing various medical abnormalities (Chen *et al* 2020a). The research utilized a three-part medical VA framework, incorporating question semantic classification, vision-based candidate answer classification, and retrieval-based candidate answer selection. It effectively integrated language and vision components, leveraging pre-trained BioBERT for question classification and ResNeSt for image classification. The model underwent training and evaluation on ImageCLEF 2020 VQA-Med datasets, achieving an accuracy score of 0.426 and a BLEU score of 0.462, securing the 4th position in the competition.

Rather than training from scratch, the next study adapted advanced pre-trained visual and language models to further enhance the question-answering capabilities in medical imaging (Wang *et al* 2023c). The research employed the BLIP-2 framework, which integrated an image encoder, query transformer (Q-Former), and LM for vision-to-language tasks. The training process consisted of two stages: initially, the image encoder and language models were frozen for vision-language representation learning, followed by a focus on vision-to-language generative learning with a frozen LM. Training took place over four days using PaddlePaddle on Ascend 910 NPUs, involving fine-tuning specific components while preserving the LM’s text generation capabilities. Evaluation on a manually split endoscopic dataset revealed that in the visual question answering task, the model exhibited high performance on the validation set, with accuracies exceeding 0.9 for most questions and notable improvements in polyp-related questions following the use of separate classification models. However, its performance on the test set was less impressive, achieving an overall accuracy of 0.7396. The model excelled in some areas but faced challenges in processing detailed visual features, particularly in questions related to the color and location of abnormalities.

Advancing the complexity of these models, the introduction of unimodal and multimodal contrastive losses (Li *et al* 2023b) marked a significant progression in aligning image and text features through self-supervised learning. The model, based on a transformer architecture with separate image, text, and multimodal encoders, underwent pre-training using medical image-caption pairs, with 25% of image patches masked and employing masked language modeling (MLM) loss. For downstream medical VQA tasks, a 6-layer transformer-based decoder generated answers with MLM loss. The approach incorporated unimodal and multimodal contrastive losses (UCL and MCL) to align image and text features, used an image text matching strategy for binary classification, and employed a masked image strategy for data augmentation. This model surpassed existing models with significant performance improvements, including a 2.2% increase on VQA-RAD, substantial margins on PathVQA, and a 1.7% improvement on SLAKE, enhancing both closed-ended and open-ended answer accuracy. Ablation studies confirmed the significance of UCL and MCL in improving multimodal representation learning across all medical VQA datasets.

Expanding beyond self-supervised learning techniques, the medical general-purpose vision system (MED-GPVS) (Haridas *et al* 2022) emerged as a versatile, task-agnostic model, broadening the scope of medical image interpretation and Q&A. The MED-GPVS model merged features from DETR (DEtection TRansformer) and ViLBERT (Lu *et al* 2019) architectures to create a hybrid cross-modal understanding model. It used a ResNet-50 backbone for image feature extraction and a DETR transformer encoder–decoder architecture for object detection in the vision module. The language module employed BERT for task description encoding and text response generation. The cross-modal module used ViLBERT’s co-attention layers to combine vision and language features for diverse output modalities prediction. In object detection, the model achieved its highest accuracy of 67.48% at the 200th epoch, while in visual question answering, it reached peak accuracy of 82.40% at the 100th epoch, displaying task inference capability. However, it showed some limitations in CT scans of mediastinal images, excelling in CT imaging and VQA with over 82% accuracy, but slightly lower performance in MRI and x-ray images due to class imbalances.

From the generative approach in VQA, we move to the specialized field of histopathology, where enhanced mitosis detection represented a critical advancement for cancer prognosis and treatment assessment (Ding *et al* 2023). The study utilized vision-language models, CLIP and BLIP, for improved mitosis detection in histopathology images. CLIP optimized image and text encoders for similarity, while BLIP aligned vision and language with pre-trained components. These models underwent training on large datasets and fine-tuning on MIDOG22, evaluating performance based on F1 scores and AUC across five test splits. Baseline models, including ImageNet-pre-trained ResNet-50 and ViT-B/16, were compared, with ResNet-50 showing the best results. Among vision-language models, BLIP consistently outperformed CLIP, especially after fine-tuning, highlighting the value of combining visual and language information. The inclusion of metadata improved model performance, but CLIP didn’t surpass vision-only models. The research suggests promise in using multimodal vision and language models like BLIP for mitosis detection in binary tile classification.

Finally, the development of the first pathology-specific VQA system (He 2021) showcased an innovative three-level optimization framework, setting new frontiers in cross-modal self-supervised pretraining and finetuning for pathology. This research introduced a three-level optimization framework for VQA on the PathVQA dataset, including self-supervised pretraining, VQA finetuning, and model validation stages. The model combined image and text encoders, with a focus on identifying and excluding incorrectly labeled examples during pretraining. Two established VQA methods, a Transformer-based model and a GRU-based model with bilinear attention, are employed. The results showed significant performance improvement in VQA with the three-level optimization framework applied to both Method 1 and Method 2, effectively identifying and removing noisy training examples to prevent model distortion. The adoption of cross-modal self-supervised learning, enhanced performance by facilitating semantic correspondence learning between images and text. Crucially, omitting images from the VQA models results in a substantial performance drop, emphasizing the valuable contribution of images in the dataset and the significance of the Pathology VQA dataset for VQA tasks.

The summary of publications related to visual question answering is presented in table 14. Table 15 summarizes the specific limitations and future perspectives of the reviewed papers, along with the common limitations and future perspectives discussed in this section.

**Table 14.** Overview of LMs for visual question answering. The asterisks (*) indicate terms that are either not present in the original paper or do not apply in this context.

References	ROI	Modality	Dataset	Model name	Vision model	Language model
Wang *et al* (2023c)	Gastrointestinal Tract	Endoscope	ImageCLEFmedical2023	BLIP-2	ViT-g/14	GLM-6b
Chen *et al* (2020a)	*	*	ImageCLEF 2020 VQA-Med, VQA-Med-2019 Dataset	*	ResNet-34, Bilateral-branch network (BBN), ResNeSt-50	BioBERT
Ding *et al* (2023)	Mitosis	Histopathology	MIDOG22	CLIP, BLIP	ViT-B/32, ViT-B/16	GPT-2
Li *et* *al* (2023b)	*	*	ROCO, MedICaT, ImageCLEF2022, VQA-RAD, PathVQA, SLAKE	*	ViT	BERT
Haridas *et al* (2022)	39 Organs	CT, MRI	SLAKE	MED-GPVS	ResNet-50, DETR	BERT, ViLBERT
Zhang *et al* (2023a)	*	x-ray, CT, MRI, Microscopy	PMC-VQA(Proposed), VQA-RAD, SLAKE	MedVInT	ResNet-50	Transformer
He (2021)	*	Histopathology	PathVQA	*	Cross-modal encoder (Method 1), Faster R-CNN (Method 2)	Transformer (Method 1), Gated Recurrent unit(GRU) (Method 2)

**Table 15.** Comparative assessment of limitations and future perspectives for research in visual question answering.

References	Specific limitation	Specific future perspective
Ding *et al* (2023)	Scope limited to binary tile classification; whole-slide mitotic density not detailed.	Exploration of more vision-language models; investigation of different prompting strategies; extension to other prediction tasks.
Zhang *et al* (2023a)	Inherent biases in PMC-VQA dataset; potential annotation biases; lacking comprehensive evaluation metrics.	Addressing dataset biases; enhancing annotation quality; exploring comprehensive evaluation metrics.
He (2021)	Restricted diversity in pre-training datasets; computational complexity and resource intensity; limited exploration of model interpretability.	Incorporate diverse datasets for robust pre-training; optimize computational demands; research model interpretability and explainability.

Table 16 assessed the shared limitations and future perspectives of the reviewed papers from sections 3.1–3.7.

**Table 16.** Assessment of shared limitations and shared future perspectives for previous studies from sections 3.1–3.7.

Application	Shared limitation	Shared future perspective
Finding extraction from radiology report	1. Complex medical language processing: challenges in accurately processing and interpreting complex medical language structures	1. Advancements in medical language processing: innovations to improve accuracy in processing and interpreting complex medical language structures
Medical image/video captioning	2. Data limitations: challenges with dataset size, diversity, and quality affecting model performance and generalizability	2. Enhanced data resources: efforts to acquire larger, more diverse, and higher-quality datasets to enhance model training, performance, and generalizability
Diagnosis interpretability	3. Model training and stability: issues with the effectiveness and specificity of chosen models or methodologies, including labeling accuracy and classifier performance	3. Model training optimization: research on refining models and methodologies, focusing on effectiveness, specificity, labeling accuracy, and classifier performance
Medical report classification	4. Model specificity and language limitations: dependency on specific data formats, models, languages, or institutional settings, which may limit generalizability and impact versatility	4. Model generalization and adaptability: developing models and approaches that are less reliant on specific data formats, languages, or settings, enhancing generalizability and versatility
Medical report generation	5. Data diversity and availability: common issues include reliance on limited datasets, affecting model training and generalization	5. Promoting data diversity: initiatives to broaden datasets, ensuring they are more representative and comprehensive, improving model training and applicability
Multimodal learning		
Medical visual question answering		

### 3.8. ChatGPT for medical imaging

**Figure 7.**
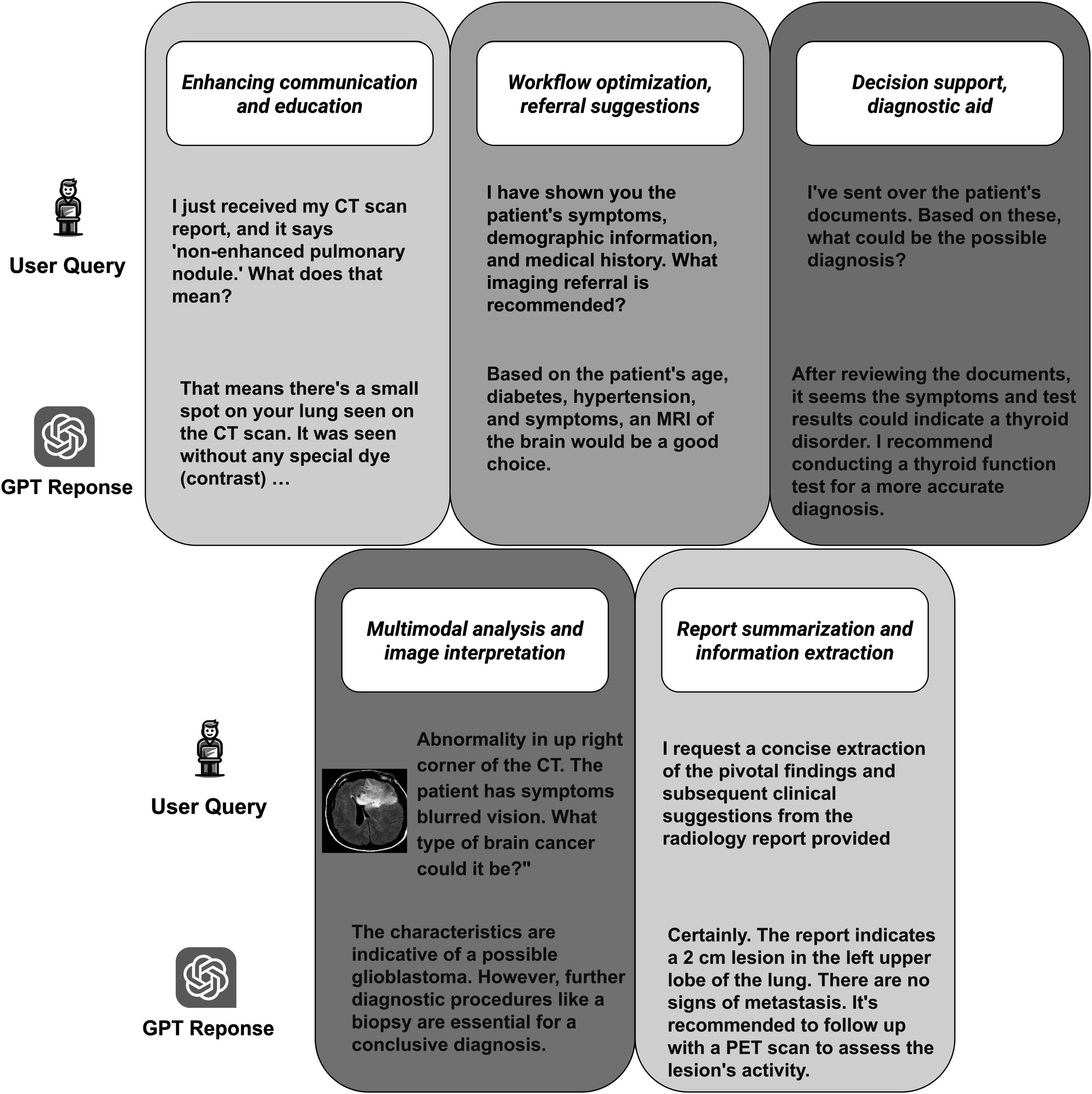
Overview of ChatGPT in various medical imaging applications. In this illustration, we have provided a simplified representation of a user query and ChatGPT’s corresponding response to facilitate a clearer comprehension of each application type for our readers.

This session initiates with a focus on ChatGPT, a transformative and advanced model that has significantly impacted the natural language processing field. The intention is to broaden knowledge of its potential utilization in medical image studies. Gifted with the competence to decipher and fabricate human-like dialogues, ChatGPT holds a vital position in the domain of visual question answering and diagnosis, where medical visuals are accompanied by clinical queries. By harnessing the power of ChatGPT, we can potentially improve the accuracy and accessibility of medical image diagnosis, particularly in remote or underdeveloped areas with limited access to expert dermatologists. The potential of ChatGPT in medical image research is still largely unexplored, but we believe that it has the potential to transform the field and revolutionize the way we approach medical imaging. Based on ChatGPT’s specific use-cases in the field of medical imaging, we have further divided this chapter into the following subsections: enhancing communication and education (section 3.8.1), workflow optimization and referral suggestions (section 3.8.2), decision support and diagnostic aid (section 3.8.3), multimodal analysis and image interpretation (section 3.8.4), along with report summarization and information extraction (section 3.8.5). To help our readers better understand the application scenarios for each subsection, as shown in figure 7, we have provided examples of questions users may pose in the prompt and potential responses that ChatGPT may provide. For the sake of user easier comprehension and due to space constraints in the figure, both the questions and responses have been simplified and are for illustrative purposes.

#### 3.8.1. Enhancing communication and education

In one of the earliest research studies that explored the use of ChatGPT to simplify radiology reports, the research assessed their quality and implications (Jeblick *et al* 2023). In a study led by an experienced radiologist, three original reports covering various medical scenarios—musculoskeletal radiology, brain neuroradiology, and oncological imaging—were simplified using a heuristic prompt in ChatGPT, resulting in 15 distinct simplified versions for each report. Radiologists evaluated these simplified reports, assessing factual correctness, completeness, and potential harm via a questionnaire featuring Likert scale ratings and free-text responses. The results indicated consensus among radiologists regarding factual correctness and completeness, with strong agreement in their assessments. However, there were varying opinions on potential harm associated with some of the simplified reports, leading to disagreements among radiologists. The study examined different cases individually, revealing similar performance across them. Overall, the findings suggest that ChatGPT shows promise in simplifying medical texts. Still, they also underscore the importance of addressing potential issues, particularly those related to interpretation and potential harm.

Building upon this exploration, the subsequent study narrows its focus, evaluating ChatGPT’s capacity to specifically lower the reading level of diagnostic radiology reports across various imaging modalities (Li *et al* 2023a). In this study, reports were categorized by anatomic region, and readability metrics such as word length, Flesch reading ease score (FRES), and Flesch-Kincaid reading level (FKRL) were employed to assess their readability. Statistical analyses were conducted to compare different report types, and reports were de-identified and simplified using ChatGPT. The study aimed to evaluate the effectiveness of ChatGPT in enhancing the readability of radiology reports, with a significance level set at 0.05. The primary findings indicated that ChatGPT significantly improved the readability of radiology reports across all modalities. Initially, a substantial proportion of original reports had low FKRL, with considerable variations among modalities. However, after ChatGPT simplification, all reports achieved FKRL values below 8.5, with 77% of XR, 76% of US, 65% of CT, and 58% of MRI reports achieving FKRL values below 6.5.

Expanding the analysis, another study undertakes a comparative review of various large language models, including ChatGPT, Google Bard, and Microsoft Bing, examining their effectiveness in simplifying radiology reports (Doshi *et al* 2023). A dataset of 254 anonymized reports from the MIMIC-III database was used, and three distinct prompts were applied for simplification. Each LLM individually processed the reports, generating simplified versions. Readability was evaluated using established indices, which were averaged to calculate an overall reading grade level score (aRGL). Statistical tests were conducted for comparison. The findings consistently demonstrated that ChatGPT-3.5 and ChatGPT-4.0 outperformed other models, achieving the lowest median aRGL scores across various imaging modalities and prompts. In particular, ChatGPT-3.5 exhibited the lowest median aRGL outputs with Prompt 2 and Prompt 3, showcasing its consistent excellence in simplifying radiology reports, with notably lower aRGL scores, making it a promising tool for improving patient health literacy and communication in medical imaging.

Further exploring the advances in different languages, a study on GPT-4-v reveals its significant performance in medical education and assessment with Spanish (Guillen-Grima *et al* 2023). The evaluation of GPT’s performance in the Spanish medical residency entrance examination (MIR) assessed its ability to answer 182 multiple-choice questions across various medical specialties in both Spanish and English. GPT-4-v excelled in this task, achieving an impressive 86.81% accuracy in Spanish, a significant improvement over GPT-3.5. The model consistently performed well across two submission attempts and showed nonrandom question sequencing patterns. Question length did not impact its performance, but it faced challenges with image-based questions, achieving success rates of 13.0% in Spanish and 26.1% in English. Importantly, no errors resulted in patient harm or death during the evaluation.

While advancements in language models show promise, it is also crucial to examine their application critically, as highlighted by a study (McCarthy *et al* 2023) evaluating ChatGPT’s use as a patient education tool, revealing areas in need of improvement. The study’s evaluation of ChatGPT found that its content was longer and more complex than the society of interventional radiology (SIR) website, with generally challenging readability levels exceeding recommendations. While ChatGPT provided comprehensive answers in many cases, it had 12 incorrect outputs out of 104 queries. When assessed for understandability and actionability, ChatGPT’s content scored lower than the website material using the PEMAT-P tool. Limitations include potential bias in accuracy assessment, the tool’s inability to score chatbot content without visual aids, and the lack of constraints on chatbot output, leading to longer responses. The study underscores the challenges of relying on AI-driven chatbots for healthcare information, emphasizing concerns about accuracy, unclear sources, and ethics. Future research is suggested to explore the utility of generative AI for patient education, focusing on content understandability and the potential for developing customized chatbots to address limitations.

Finally, a broader look at the use of ChatGPT in radiology reporting acknowledges the promising potential of these technologies in enhancing patient engagement, while also considering the challenges they face. ChatGPT’s potential applications in radiology include automating clinical history sections in reports, enhancing report impressions, generating layperson reports in multiple languages, prompting meaningful patient-provider interactions, and aiding quality control. However, limitations such as biases, data privacy, interpretability issues, vulnerability to adversarial attacks, and ethical concerns exist. The future direction emphasizes meticulous integration and validation of LLM outputs, particularly for abstract reasoning and contextual medical understanding tasks.

#### 3.8.2. Workflow optimization and referral suggestions

As the first investigation (Lim *et al* 2023) into the accuracy and limitations of AI chatbots, specifically ChatGPT, in providing information about standard GI endoscopic procedures, the research involved querying ChatGPT about these procedures, assessing its accuracy in providing details, alternatives, risks, and risk quantification. Three iterations were performed for each set of questions to examine variations. Evaluation criteria encompassed factual accuracy, comprehensiveness, safety concerns, internal consistency, and linguistic fluency. Three authors collectively assessed ChatGPT’s responses, with individual observations aggregated for analysis. The study found that ChatGPT could offer easily comprehensible advice for laypersons. Information about periprocedural care and specific procedures like EGD, colonoscopy, and EUS, mainly for diagnostic purposes, was of acceptable quality with minor inconsistencies. However, it is crucial to note that information regarding ERCP contained significant inaccuracies and errors, which varied across different iterations.

Moving to a comparative analysis where the capabilities of ChatGPT and other LLMs were juxtaposed against expert neuroradiologists in neuroimaging recommendations, the study used prompts based on ACR appropriateness criteria (AC) variant titles, customized for a 65 year-old patient as input for LLMs (Nazario-Johnson *et al* 2023). ChatGPT ranked the top three modalities, while Glass AI received the same prompts without ranking. Evaluation involved two authors scoring the outputs based on predefined guidelines, considering specificity with penalties for nonspecific responses, and a neuroradiologist providing single best modality selections. Statistical analysis, including the Wilcoxon signed rank test with a Bonferroni correction, was conducted to compare LLM and neuroradiologist performance. ChatGPT achieved a mean score of 1.75, Glass AI scored slightly higher at 1.83, and the neuroradiologist outperformed both with a mean score of 2.20 in recommending radiologic imaging modalities. Although ChatGPT and Glass AI performed similarly, the difference was not statistically significant (*P* = 0.31). These findings suggest potential for ChatGPT’s improved performance with medical text training but highlight the continued need for enhancement in the medical context, as LLMs did not surpass the experienced neuroradiologist’s performance.

Building on the comparative insights, the focus then shifts to the development of accGPT (Rau *et al* 2023), illustrating a practical application of AI in providing personalized imaging recommendations. The study utilized 209 topics from ACR criteria as foundational knowledge, extracting text information from ACR guidelines and encoding it into data nodes using an embedding model indexed with LlamaIndex. Chatbot responses were tailored for case-based scenarios and evaluated by six radiologists using 50 clinical case files. Chatbots, including accGPT, matched or exceeded human radiologists’ performance, with AccGPT providing 83% ‘usually appropriate’ recommendations, outperforming radiologists at 66%, while GPT 3.5-Turbo achieved 70%, and GPT 4 reached 79% accuracy. AccGPT exhibited high consistency with a Fleiss’ Kappa of 0.82. Additionally, the chatbots significantly reduced decision time (5 min) and costs (0.19 Euro) compared to radiologists (50 min and 29.99 Euro, respectively), with both differences being statistically significant (*p* < 0.01).

Expanding the application of AI in medical referrals, the next study delves into the potential of ChatGPT-4 for automating the radiology referral process in emergency department settings (Barash *et al* 2023). In a retrospective study, ChatGPT-4 was evaluated for generating referral notes from clinical admission notes. Two radiologists assessed the generated notes for clarity, clinical relevance, and accuracy, comparing them with actual ED examinations and ACR AC guidelines. GPT-4 exhibited perfect alignment with ACR AC and ED examinations in all 40 cases, indicating a high level of concordance. Independent reviewers provided favorable ratings, with mean scores ranging from 4.4 to 4.9 out of 5 for clarity, clinical relevance, and differential diagnosis. The agreement between reviewers was moderate to substantial, reflecting reliable performance. Minor discrepancies included protocol variations in two cases and occasional omissions related to symptom onset and final diagnoses in a small fraction of cases, although these were relatively infrequent.

Further exploring the capabilities of different versions of ChatGPT, a comparative evaluation highlighted the advancements in accuracy and clinical utility, particularly in the context of radiologic decision support. The study found that for breast cancer screening prompts, both ChatGPT-3.5 and ChatGPT-4 achieved high average OE scores of approximately 1.83 out of 2, but ChatGPT-4 demonstrated significantly better accuracy with an average percentage correct of 98.4%, compared to ChatGPT-3.5’s 88.9%. In the context of breast pain, ChatGPT-4 also outperformed ChatGPT-3.5, with higher average OE scores (1.666 versus 1.125) and SATA average percentage correct (77.7% versus 58.3%).

Finally, we conclude this subsection with an investigation into the broader reliability of ChatGPT as a resource for imaging referrals, probing its potential to enhance healthcare decision-making processes. The study (Rosen and Saban 2023) conducted a two-stage analysis comparing ChatGPT’s performance in providing accurate clinical imaging referrals with the ESR iGuide, based on GPT-3 and ACR guidelines, respectively, involving 97 patient cases in the emergency department. A dichotomic rating system determined appropriateness, and specialists evaluated ChatGPT’s recommendations in a sub-group analysis. ChatGPT showed substantial agreement of 87.6% with ESR iGuide recommendations, scoring between 7 and 9 on the appropriateness scale in 87.4% of cases, with age and gender having no significant impact on this agreement. This research highlights the potential use of language models in healthcare decision-making, particularly in emergency imaging referrals.

#### 3.8.3. Decision support and diagnostic aid

The first study (Horiuchi *et al* 2024) begins by investigating ChatGPT’s performance in neuroradiology, filling a significant knowledge gap and highlighting its potential to enhance diagnostic accuracy in this specialized field. The evaluation of ChatGPT for diagnosing neuroradiology cases involved utilizing patient information, including medical history and imaging findings, as input data. The study aimed to assess ChatGPT’s diagnostic accuracy by generating differential diagnoses and a final diagnosis based on this input. Diagnostic accuracy rates were used as the primary metric, compared across various anatomical locations and disease categories. The dataset included 100 consecutive neuroradiology cases with detailed patient medical histories and imaging findings extracted. ChatGPT was employed to analyze and interpret this data for each case individually, avoiding bias from previous answers. Experienced neuroradiologists then evaluated ChatGPT’s diagnoses against the actual ground truth. Using Fisher’s exact test, comprehensively assessed ChatGPT’s diagnostic performance across different anatomical locations and disease categories. This methodology effectively addressed the research objectives, providing valuable insights into ChatGPT’s capabilities in interpreting complex patient data in neuroradiology.

The final diagnostic accuracy was 50%, with a differential diagnostic accuracy of 63%. ChatGPT may be utilized regardless of anatomical locations in neuroradiology. However, the diagnostic accuracy may vary depending on disease etiologies. For instance, CNS tumor cases exhibited significantly lower accuracy rates compared to non-CNS tumor cases. Disease etiologies within the non-CNS tumor group also showed varying accuracy levels, with metabolic, cerebrovascular, and degenerative diseases having relatively higher accuracy. However, inflammatory diseases had a lower final diagnostic accuracy rate. These findings emphasize ChatGPT’s potential in neuroradiology but highlight the need for further optimization, especially in complex cases with varying disease etiologies.

From the specific use of ChatGPT in neuroradiology, the focus shifts to a broader assessment of ChatGPT-3’s accuracy in answering radiologists’ questions across various domains, evaluating its ability to provide accurate and relevant references. GPT-3 was assessed by presenting 88 questions from various radiology subspecialties. Responses were evaluated for accuracy using a 5-point scale, and the model’s reference-providing capability was examined. Out of 88 questions, ChatGPT-3 provided correct responses to 67%, partially correct responses to 17%, and had varying degrees of incorrect responses for the rest. When asked to provide references, it generated 63.8% of references, while the rest were real references. Among the real references, 82.3% were indexed in PubMed. ChatGPT-3 showed reasonable accuracy in answering questions and providing references but had some issues with generating references.

Different from previous studies, the next one explored the role of ChatGPT in facilitating the development of deep learning models for detecting bone metastasis, addressing the convergence of medical and programming expertise (Son *et al* 2023). ChatGPT played a vital role in aiding a physician with limited programming experience in developing and optimizing a deep learning model. It assisted in generating the initial code, ensuring its functionality, and provided guidance throughout the optimization process. With ChatGPT’s help, the model achieved optimal performance with a batch size of 16 and an epoch size of 150, resulting in a low test loss of 0.2894 and a high AUC of 0.8156. This model demonstrated excellent diagnostic capabilities, with a sensitivity of 56% and a specificity of 88.73%, particularly in the detection of bone metastasis in bone scans across various malignancies.

#### 3.8.4. Multimodal analysis and image interpretation

The first research (Buckley *et al* 2023) represented an initial investigation into the accuracy of a GPT-4-V on a wide range of challenging medical cases. The authors compiled a dataset with 936 medical cases from the NEJM Image Challenge, spanning 2005 to October 2023, and utilized 80 NEJM clinicopathological conferences from January 2021 to December 2022. GPT-4-V model experiments were conducted in October 2023, with precautions against information leakage. The study evaluated GPT-4-V’s responses against reference answers, achieving an impressive overall accuracy of 61%, surpassing human respondents’ 49% accuracy across challenging medical cases. The model consistently outperformed humans across difficulty levels and disagreements among respondents, excelling in cases with informative text descriptions. GPT-4-V’s performance was consistent across skin tones and image types, except for radiographic images, where it performed similarly to humans. In clinicopathological conferences, it included the correct diagnosis in the differential for 30% of cases in the multimodal setting and 32% in the text-only setting.

Building upon this initial exploration, the next study became the first to publicly benchmark GPT-4-V’s performance, specifically in tasks like radiology report generation and medical VQA (Li *et al* 2023c). The evaluation of GPT-4-V encompassed Radiology Report Generation, Medical Visual Question Answering, and Visual Grounding tasks, with metrics like accuracy, relevance, and conciseness. Datasets used included MIMIC-CXR, VQA-RAD, and MS-CXR. GPT-4-V performed well in Radiology Report Generation, accurately creating reports from medical images. In Medical Visual Question Answering, it provided relevant responses to medical queries. However, in Visual Grounding tasks, its performance varied, with some difficulty in precisely identifying and localizing medical conditions and organs in images.

Expanding the scope of evaluation, Wu *et al* (2023) conducted a comprehensive assessment of GPT-4-V across various medical modalities, highlighting the model’s current limitations and challenges in real-world applications. The evaluation of GPT-4-V encompassed tasks related to radiology image recognition, diagnosis, and report generation using Radiopaedia, as well as pathology image analysis using PathologyOutlines. Public medical image benchmarks were used, considering factors like release time, annotation reliability, and diverse imaging modalities. Image processing rules and question prompts were applied for evaluation, with reference captions from Radiopaedia for comparison. GPT-4-V demonstrated proficiency in distinguishing between image modalities and anatomy but faced challenges in disease diagnosis and generating comprehensive medical reports.

Focusing on a specific medical application, the subsequent research assessed GPT-4-V’s capabilities in analyzing ocular images, considering the impact of clinical context inclusion (Sorin *et al* 2023). In a retrospective evaluation of GPT-4-V’s diagnostic performance on ocular images, a dataset of 40 anonymized ocular images was used, representing various conditions. The study compared GPT-4-V to two non-ophthalmologist physicians in both image-only and image with clinical context scenarios. GPT-4-V initially achieved a diagnostic accuracy of 47.5% without clinical context, while physicians achieved 60.0% and 57.5% accuracy. With clinical context, GPT-4-V’s accuracy improved to 67.5%, matching physicians closely. No statistically significant difference was observed between GPT-4-V and physicians, emphasizing the value of clinical context in enhancing diagnostic accuracy, with GPT-4-V showing potential in ophthalmology diagnosis, especially with context-rich information.

Adding to the comprehensiveness of GPT-4-V evaluations, Chen *et al* (2023) thoroughly assessed its proficiency across a broad spectrum of medical imaging modalities and question types, further establishing its versatility. A direct comparison was conducted with traditional ResNet models in classifying COVID-19 images, focusing on accuracy and efficiency. GPT-4-V’s best performance was an F1 score of 0.85 for COVID images and an accuracy of 0.85, outperforming traditional models like ResNet-18 and VGG16 in few-shot scenarios but falling short of their performance when fully trained, highlighting its potential and need for optimization.

Exploring an ambitious application, Aydin (2023) delved into the potential of GPT-4-V to detect diseases and function as a standalone medical practitioner, pushing the boundaries of AI in healthcare. The study used a chest film database created during the COVID-19 pandemic, processed images one by one with GPT-4, and examined the model’s comments. Additionally, the research explored combining multiple pictures into one image. The implementation of ChatGPT with the images gave promising results.

Finally, the zero-shot detection of changes in MRI brain images using GPT-4-V underlined the model’s capacity for detailed and time-sensitive analysis (Kelly *et al* 2023). Patients imaged between 2019 and 2022 were included. A binary classification task using accuracy as the primary metric, with a focus on identifying misclassifications, was conducted. Each case was evaluated in separate chat sessions to ensure an unbiased assessment. The evaluation utilized a dedicated web interface for GPT-4-V, with two co-registered FLAIR MRI brain images of MS patients at different time points. Baseline models were implemented in PyTorch with data augmentation. GPT-4-V achieved an accuracy of 85% in identifying radiologic progression in MRI brain images of Multiple Sclerosis patients. This performance, while noteworthy, falls slightly short compared to the UNet and ViT models, both of which attained a higher accuracy of 94%. GPT-4-V displayed cautious answers in only a limited number of cases. The error pattern for GPT-4-V resembled the ViTs’, with a mix of false positives and false negatives, while the UNet primarily had false negatives.

#### 3.8.5. Report summarization and information extraction

Improving communication between radiographers and physicians through more effective and automated impression generation, one study (Jiang *et al* 2023) introduced a ChatGPT-based Contrastive Learning (CCL) approach for Automatic Impression Generation (AIG) from radiology reports. The method involved training the model on diverse radiology report datasets, utilizing a two-phase approach, including a warm-up stage with the T5 model and a contrastive learning phase with self-generated impressions. ChatGPT played a crucial role in data augmentation, particularly for underrepresented cases. The model’s strong performance in summarizing radiology reports is evident through its impressive ROUGE scores, with scores of 67.29, 57.48, and 67.16 on the OPEN-I dataset and 49.63 and 34.81 on the MIMIC-CXR dataset for ROUGE-1 and ROUGE-2, respectively.

The next study explored ChatGPT’s capacity to summarize complex radiologic reports int simple versions, aiming to enhance patient understanding of MRI results (Schmidt *et al* 2024). In a two-stage assessment of ChatGPT’s effectiveness in simplifying knee joint MRI reports, medical professionals, including experienced orthopedic surgeons and radiologists, evaluated the quality, completeness, and comprehensibility of simplified reports in the first stage. In the second stage, 20 patients provided feedback on reports of varying complexities. Medical professionals generally found the simplified reports factually correct and comprehensible, although concerns emerged with complex reports. Patients found the reports simple and comprehensible, but complex reports had lower information content ratings. While ChatGPT aids communication, it doesn’t replace the expertise of medical professionals.

**Figure 8.**
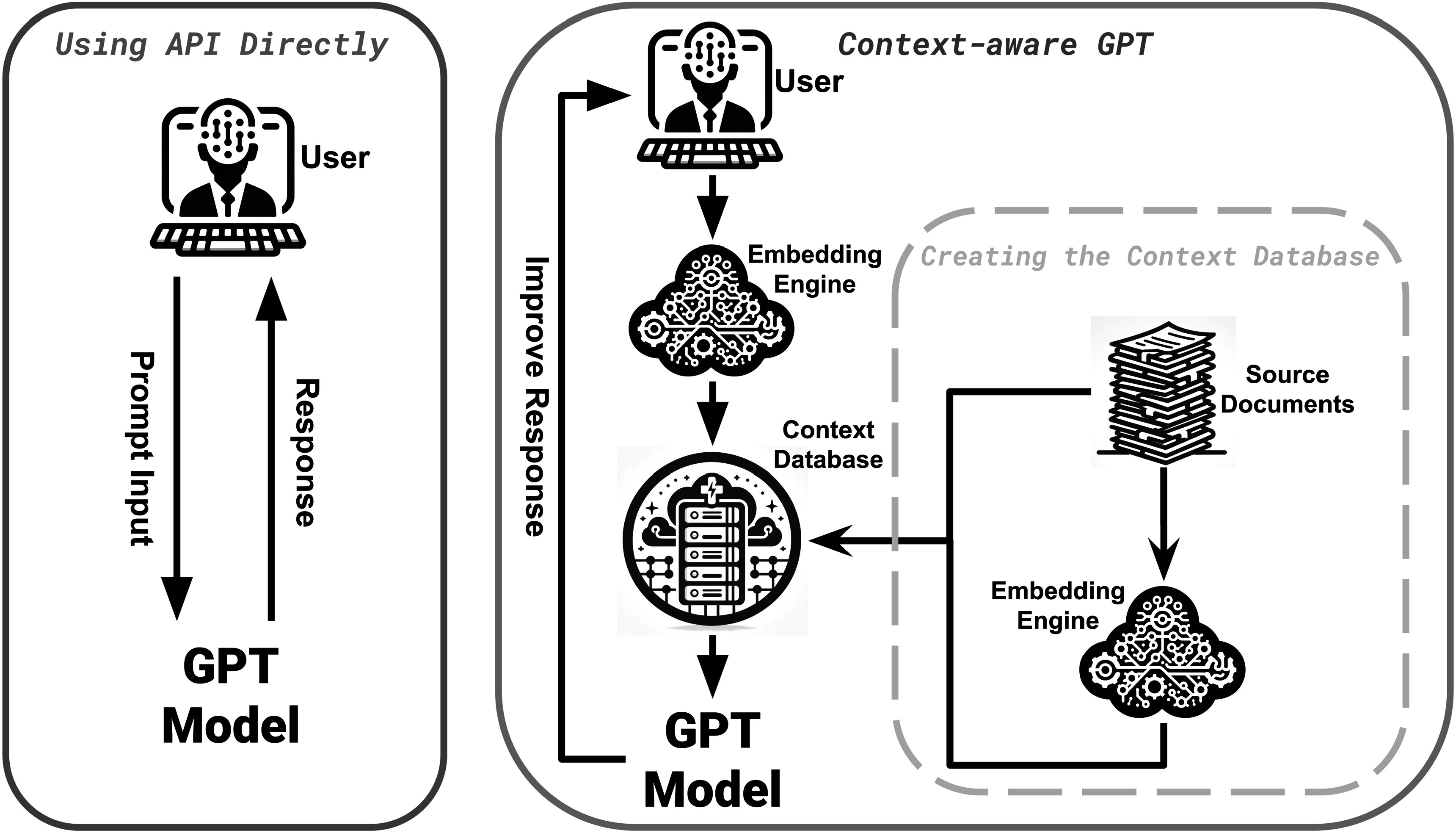
Comparison of using the API directly versus building a context-aware chatbot.

From patient-focused report summarization, we move to a more specialized application, where ChatGPT was integrated into a context-aware chatbot (Russe *et al* 2023) for accurate fracture classification using radiology reports. The process of building a context-aware chatbot is shown in figure 8. The evaluation of chatbots, including the context-aware FraCChat, for fracture classification based on radiological reports, incorporated specific knowledge from the AOOTA Fracture and Dislocation Classification Compendium—2018 using the LlamaIndex interface. A dataset of 100 radiological reports was used to compare chatbots and human radiologists in accuracy, consistency, and efficiency for fracture classification. Human radiologists achieved 95% accuracy in providing correct full AO codes, surpassing generic chatbots (GPT 3.5-Turbo and GPT 4) with only 5% and 7% accuracy, respectively. Context-aware chatbots, particularly FraCChat based on GPT 4, showed significant improvements, reaching 83% accuracy for full AO codes and matching human performance in some aspects. Context-aware chatbots also offered more consistent answers, especially in type and subgroup classification. Chatbots proved to be more time-efficient than human readers, with generic chatbots being faster than context-aware ones, and GPT 3.5-based chatbots being quicker than GPT 4-based ones.

Lastly, delving into a unique application, research on synchronous bilateral breast cancer utilized ChatGPT to generate a detailed case report (Naik *et al* 2023), showcasing its potential in extracting information from specific and nuanced medical scenarios. ChatGPT’s capability in generating a medical case report was evaluated using perplexity and burstiness as key metrics, comparing the AI-generated text against both human-authored and other AI-generated reports. This quantitative assessment was complemented by a qualitative review. The results indicated a lower perplexity and burstiness for ChatGPT-generated content compared to human-authored text, suggesting better coherence and sentence variation. Across three versions of ChatGPT queries, the perplexity ranged from 35 to 44, and burstiness from 15.7 to 22. In contrast, a human-authored report showed a higher perplexity of 120.3 and burstiness of 88.8. The final mixed human-AI manuscript achieved a middle ground with a perplexity of 62.3 and burstiness of 45.8, indicating that while ChatGPT excelled in linguistic fluency, the optimal application of its outputs benefits from human collaboration and review.

The summary of publications related to ChatGPT for medical imaging is presented in table 17.

**Table 17.** Overview of GPTs for medical imaging applications. The asterisks (*) indicate terms that are either not present in the original paper or do not apply in this context.

References	ROI	Modality	Dataset	GPT version
Li *et al* (2023c)	*	x-ray	MIMIC-CXR, VQA-RAD, MS-CXR	GPT-4-V
Rau *et al* (2023)	*	*	Private case file, ACR protocol	GPT-3.5-Turbo, GPT-4
Buckley *et al* (2023)	Skin, eyes, mouth	Radiographic images, ocular images, oral images	NEJM image challenge cases, NEJM clinicopathological conferences	GPT-4-V
Horiuchi *et al* (2024)	Rain, spine, head and neck	*	‘Case of the Week’ by American Journal of Neuroradiology	GPT-4
Wagner and Ertl-Wagner (2023)	Neuro, pediatrics, gastrointestinal, genitourinary, cardiac, musculoskeletal, breast, chest wall and pulmonary	*	88 Questions	GPT-3
Wu *et al* (2023)	17 human body systems	x-ray, CT, MRI, PET, DSA, mammography, ultrasound, pathology	Online samples from 17 human body systems	GPT-4-V
Lim *et al* (2023)	Gastrointestinal tract	EGD, colonoscopy, ERCP, EUS	*	GPT-3
Jeblick *et al* (2023)	Knee, head	CT, MRI	Fictitious radiology reports	GPT-3.5
Barash *et al* (2023)	*	*	Institutional clinical notes	GPT-4
(Son *et al* 2023)	Skeletal system	Bone Scan	Institutional bone scans	GPT-3.5
(Jiang *et* *al* 2023)	Chest	x-ray	OPEN-I, MIMIC-CXR	GPT-3
Li *et al* (2023a)	Abdomen, pelvis, neurological, musculoskeletal	x-ray, ultrasound, CT, MRI	Institutional data (400 radiology report)	GPT-3
Rao *et al* (2023a)	Breast	*	*	GPT-3.5
Rao *et al* (2023b)	Breast	*	*	GPT-3.5, GPT-4
Guillen-Grima *et al* (2023)	*	*	Questions from spanish medical residency entrance examination (MIR)	GPT-3.5, GPT-4
Rosen and Saban (2023)	*	*	Institutional 97 patient cases	GPT-3
McCarthy *et al* (2023)	*	*	Material from society of interventional radiology (sir) patient center website	GPT-3
Sorin *et al* (2023)	Eyes and ocular structures	Ocular imaging	40 institutional ocular images	GPT-4-V
Chen *et al* (2023)	Lung	x-ray	Kaggle COVID-19 Lung x-ray dataset	GPT-4-V
Yan *et al* (2023b)	Fifteen object of interest	11 modalities including microscopy, dermascope, x-ray, CT, ECHO, angiography, ultrasound, MRI, PET	VQA-RAD, PMC-VQA, PathVQA	Kaggle COVID-19 lung x-ray dataset
Li *et al* (2023a)	Chest	x-ray	Chest film database	GPT-4-V
Russe *et al* (2023)	Bone fracture	*	AOOTA fracture and dislocation classification compendium	GPT-3.5, GPT-4
Elkassem and Smith (2023)	*	*	*	GPT-3.5
Schmidt *et al* (2024)	Knee joint	MRI	*	GPT 3.5
Kelly *et al* (2023)	Brain	MRI	Institutional dataset for multiple sclerosis	GPT-4-V
Naik *et al* (2023)	Breast	Mammography, ultrasound, MRI	Institutional data of bilateral breast cancer	GPT-3.5
Lyu *et al* (2023)	Brain	CT, MRI	Atrium health wake forest baptist clinical database	GPT-3, GPT-4
Nazario-Johnson *et al* (2023)	*	*	Presentation of a 65 year-old patient	GPT-3.5
Doshi *et al* (2023)	*	MRI, CT, ultrasound, mammogram	*	GPT-3, GPT-4

## 4. Discussion

In our comprehensive literature search, we employed a meticulously crafted search query on Google Scholar and PubMed, specifically (‘Language Model’ OR Chatbot) AND (Medical OR CT OR MR OR Ultrasound OR x-ray OR OCT OR Pathology) AND (Image OR Imaging). We rigorously eliminated duplicate entries, focused exclusively on papers published from 2020 onwards to maintain relevance, and refined our criteria to include only those papers introducing novel models post-Transformer architecture, aligning with the rapidly evolving landscape of language models. As a result, our search yielded a total of 84 papers that met these stringent criteria, out of which 32 were dedicated to the exploration of ChatGPT within the context of medical imaging.

These works demonstrated the significant performance of the LMs in medical imaging tasks. Firstly, the LMs can be applied for medical image/video captioning. Researchers have developed LMs to integrate visual and textual information to generate informative captions for medical images and videos, achieving state-of-the-art performance. The LMs allow end-to-end deep learning methods to generate diagnostic reasons for their prediction, improving the interpretability of the networks. Furthermore, LMs can be applied to generate accurate and readable reports from various types of medical images to improve clinical workflow efficiency, reduce diagnostic errors, and assist healthcare professionals in providing timely and accurate diagnoses. LMs also demonstrated their application in visual question answering, patient/medical student education, pre-diagnosis screening, protocol determination, and more.

Although LMs have achieved remarkable performance in the fields mentioned above, reaching a level that can rival (Buckley *et al* 2023, Rau *et al* 2023) or even surpass (Naik *et al* 2023, Russe *et al* 2023) human, it is undeniable that LMs are far from being able to fully automate the medical imaging process, let alone operate without human expert supervision. To ensure that our readers can effectively and responsibly utilize these LMs as tools, it is essential for us to discuss the limitations of existing LMs for medical imaging methods and the potential risks they may pose in this context. Additionally, we will examine and provide insights into possible future directions for development in this field.

### 4.1. Data quality and accessibility

In the context of LMs for medical imaging, the foremost concerns revolve around data quality and data accessibility. The efficacy of these models is heavily contingent on the caliber of the data used for training, encompassing not just data accuracy and relevance but also its capacity to encompass a wide array of medical scenarios. High-quality data is instrumental in ensuring precise and dependable model predictions, a prerequisite for informed clinical decision-making. Nonetheless, the medical field frequently grapples with data quality challenges stemming from issues such as inconsistent annotation, variations in medical imaging protocols, and the presence of artifacts. Simultaneously, data accessibility presents another formidable obstacle. Stringent privacy regulations and ethical considerations pertaining to patient data substantially restrict the availability of expansive and varied datasets. This constraint curtails the capacity of language models to learn from a diverse spectrum of clinical cases, thereby impacting their ability to generalize and perform effectively in practical applications. Consequently, the development of robust LMs for medical imaging demands a concerted endeavor to elevate both the quality and accessibility of medical data. This may encompass the establishment of standardized protocols for data collection and annotation, fostering collaborative data sharing initiatives that respect privacy, and implementing techniques like synthetic data generation to mitigate the challenges posed by the scarcity of real-world data. Effectively addressing these issues is pivotal for unlocking the full potential of LMs in reshaping medical imaging and advancing patient care.

### 4.2. Privacy and security concerns

The integration of language models in medical imaging raises significant concerns regarding privacy and security. These concerns are twofold: the protection of sensitive patient data and the security of the models against malicious use.

Privacy concerns primarily stem from the need to handle sensitive patient information, including medical histories, diagnostic images, and personal identifiers. The use of this data in training and operating language models poses risks, such as unauthorized access or data breaches, leading to potential violations of patient confidentiality. These risks are exacerbated by the large-scale data requirements of language models, which often necessitate aggregating data from multiple sources, increasing the likelihood of exposure. Moreover, the potential for residual information from training data to be inferred from the model’s outputs adds another layer of privacy challenges. Ensuring compliance with regulations like HIPAA in the U.S. or GDPR in Europe is crucial for maintaining patient trust and legal conformity.

Security concerns are equally critical. The materials indicate a risk of adversarial attacks (Qiu *et al* 2019), where malicious inputs are designed to deceive the model into making incorrect predictions or revealing sensitive information. In the context of medical imaging, this could mean manipulating an image to mislead a diagnosis or extracting patient data embedded in the model. The increasing sophistication of these attacks necessitates robust defense mechanisms to ensure the integrity and reliability of the models.

Addressing these privacy and security challenges requires a multifaceted approach. For privacy, implementing stringent data anonymization techniques and secure data handling protocols is essential. Regular audits and compliance checks can also ensure ongoing adherence to privacy standards. For security, incorporating advanced adversarial defense mechanisms in the model’s architecture and continuous monitoring for potential threats are necessary. Additionally, fostering a culture of ethical AI use and implementing strong legal frameworks will be crucial in safeguarding against misuse and maintaining public trust in these advanced technologies.

### 4.3. Model interpretability and blackbox problem

Medical imaging, such as MRI, CT scans, or x-rays, involves complex visual data that can be challenging to interpret even by trained professionals. When language models like ChatGPT are employed in this context, the interpretability of these models is crucial because it directly impacts clinical decision-making and patient outcomes. The ‘black box’ (Mannarswamy and Chidambaram 2021) nature of models like ChatGPT becomes a significant hurdle here. Understanding the basis on which these models draw such conclusions is often not straightforward. This lack of transparency can be a major barrier in clinical settings where the rationale behind each decision can be as crucial as the decision itself.

To bridge this gap, there is a need for more interpretability in medical imaging. This could involve integrating medical vision models with LMs in a way that allows for traceable and understandable decision-making processes (Tanida *et al* 2023, Yan *et al* 2023a). For instance, combining image recognition models that provide annotated images highlighting areas of interest with language models that can explain these annotations in a clinically relevant context. Additionally, developing methods for these models to provide reasoning in a manner akin to clinical thought processes can enhance their utility and trustworthiness.

### 4.4. Human collaboration and expert supervision

LMs’ effectiveness may substantially increase when coupled with the expertise of medical professionals (Tanida *et al* 2023, Wang *et al* 2023b). In the nuanced and high-stakes field of medical imaging, the knowledge and experience of clinicians, radiologists, and medical physicist are critical in guiding and refining the outputs of LMs. Human oversight ensures the contextual accuracy of LM interpretations, aligning them with the intricacies of individual patient cases and the broader clinical picture. This collaboration is crucial for mitigating potential errors, biases, or oversights that might arise solely from automated processes. Moreover, human experts play a pivotal role in integrating LM insights into practical clinical decision-making, ensuring that the technology is harnessed responsibly and ethically. Thus, the synergy between LMs and human expertise in medical imaging not only enhances diagnostic accuracy and efficiency but also upholds the standards of patient care and safety.

### 4.5. Public availability

When a powerful language model like ChatGPT is not publicly available and remains a proprietary product of a company, it influences both the accessibility and the direction of research and development in medical imaging. On one hand, restricted access to such advanced models could limit innovation in medical imaging, as researchers and clinicians might not have the opportunity to experiment with, adapt, and integrate these models into their work. This limitation could slow down the pace of discovery and implementation of new, AI-enhanced diagnostic and analytical methods. On the other hand, the proprietary nature of these models can also drive quality and safety, as the companies behind them invest in extensive research, development, and testing to ensure their reliability and efficacy. Furthermore, proprietary models might have more robust governance and ethical frameworks, as companies are accountable to regulatory bodies and stakeholders. However, this can lead to a concentration of technological advancement within certain entities, potentially creating disparities in the field. The ideal scenario may involve a balance between proprietary and open-source models, where proprietary models ensure quality and reliability, while open-source alternatives promote wider accessibility and collaborative innovation in the medical imaging community.

### 4.6. Biases and hallucination

Model biases in LMs arise from the data they are trained on, which may contain inherent biases related to demographics such as race, gender, or socio-economic status. These biases can be inadvertently transferred into the models’ predictions and analyses, potentially leading to skewed or unfair outcomes in medical diagnostics and treatment recommendations. For example, a language model trained predominantly on data from a specific demographic might underperform or yield less accurate predictions for patient populations outside that demographic. This scenario highlights the need for inclusive and diverse training datasets in the development of LMs for medical imaging. Hallucinations, another challenge, refer to instances where LMs generate plausible yet inaccurate or misleading information (Sallam 2023). This aspect is particularly concerning in medical settings, where factual accuracy is paramount. Such inaccuracies might arise due to the LM’s design to prioritize cohesive language generation over factual correctness. For instance, an LM might generate a coherent but medically inaccurate interpretation of an imaging report, leading to potential misdiagnoses. Both biases and hallucinations underscore the necessity for rigorous validation, continuous monitoring, and the involvement of medical experts in the application of LMs in medical imaging, ensuring that the outputs are not only plausible but also clinically accurate and unbiased.

### 4.7. Finetuning and domain-specific models

Fine-tuning, a process where pre-trained models like GPT-3 or GPT-4 are further trained on specific datasets, allows for the adaptation of LMs to the nuances of medical imaging. This process tailors LMs to understand and interpret complex medical terminologies, patient reports, and imaging data, leading to more accurate and contextually relevant outcomes. For instance, models like ClinicalBERT (Yan and Pei 2022), which is fine-tuned with medical texts, demonstrate improved performance in understanding clinical documentation and patient notes. Domain-specific LMs, on the other hand, are designed from the outset with a focus on a particular area of medicine. They incorporate specialized knowledge and are trained on targeted datasets, making them inherently more adept at handling the intricacies of specific medical fields, such as radiology or pathology. Such models can profoundly impact patient diagnosis and treatment planning by providing more precise and insightful analyses of medical images and reports.

### 4.8. Lack of effective evaluation metrics

The lack of effective evaluation metrics for language models in medical imaging poses a significant challenge in assessing their practical applicability and efficacy. Current evaluation approaches often fail to capture the nuanced requirements of medical imaging tasks, focusing predominantly on generic metrics that may not reflect the model’s performance in real-world clinical scenarios. For instance, while accuracy, precision, and recall are standard metrics, they might not adequately address the complexity of medical diagnostics where understanding subtle variations in imaging is crucial. Additionally, these models are frequently assessed in controlled experimental settings or on benchmark datasets that might not represent the diversity and complexity of actual clinical cases, including rare conditions or diverse patient demographics. This gap in evaluation can lead to an overestimation of a model’s readiness for clinical deployment, potentially overlooking critical shortcomings such as its ability to handle multimodal data or its performance across different demographics. Hence, there is a pressing need for developing more comprehensive and clinically relevant benchmarks that encompass a broader spectrum of medical imaging challenges. These benchmarks should ideally include diverse patient populations, varied medical conditions, and real-world clinical scenarios, offering a more holistic assessment of a model’s capabilities and limitations.

## 5. Conclusion

In conclusion, this paper has highlighted the potential of language models in advancing medical imaging analysis. We have discussed the challenges and opportunities in using language models for healthcare applications and provided an educational tutorial for researchers in this field. Our hope is that this paper will serve as an inspiration for researchers to innovate and develop new approaches to using language models to improve medical imaging analysis. We encourage researchers in the medical imaging domain to draw insights from this review and use it as a launchpad for further exploration of the possibilities of language models in healthcare. With continued research and development, we believe that language models will play an increasingly important role in improving patient outcomes and advancing medical research.

## Data Availability

No new data were created or analysed in this study.

## Disclosures

The authors declare no conflicts of interest.
